# Supplementary material for: The effect of comprehensive intervention for childhood obesity on dietary diversity among younger children: Evidence from a school-based randomized controlled trial in China
Source: PLoS One. 2020 Jul 17;15(7):e0235951. doi: 10.1371/journal.pone.0235951 (PMC7367455; doi:10.1371/journal.pone.0235951)
Supplement: S1 Checklist — (DOCX) [file pone.0235951.s002.docx]

# **S1 CONSORT checklist**

|  |  | Reporting Item | Page Number |
| --- | --- | --- | --- |
| **Title and Abstract** |  |  |  |
| Title | [#1a](https://www.goodreports.org/consort/info/#1a) | Identification as a randomized trial in the title. | 1 |
| Abstract | [#1b](https://www.goodreports.org/consort/info/#1b) | Structured summary of trial design, methods, results, and conclusions | 3-4 |
| **Introduction** |  |  |  |
| Background and objectives | [#2a](https://www.goodreports.org/consort/info/#2a) | Scientific background and explanation of rationale | 5-6 |
| Background and objectives | [#2b](https://www.goodreports.org/consort/info/#2b) | Specific objectives or hypothesis | 6-7 |
| **Methods** |  |  |  |
| Trial design | [#3a](https://www.goodreports.org/consort/info/#3a) | Description of trial design (such as parallel, factorial) including allocation ratio. | 7-8 |
| Trial design | [#3b](https://www.goodreports.org/consort/info/#3b) | Important changes to methods after trial commencement (such as eligibility criteria), with reasons | 8 |
| Participants | [#4a](https://www.goodreports.org/consort/info/#4a) | Eligibility criteria for participants | 8 |
| Participants | [#4b](https://www.goodreports.org/consort/info/#4b) | Settings and locations where the data were collected | 7,10 |
| Interventions | [#5](https://www.goodreports.org/consort/info/#5) | The experimental and control interventions for each group with sufficient details to allow replication, including how and when they were actually administered | 8-10 |
| Outcomes | [#6a](https://www.goodreports.org/consort/info/#6a) | Completely defined prespecified primary and secondary outcome measures, including how and when they were assessed | 10-11 |
| Sample size | [#7a](https://www.goodreports.org/consort/info/#7a) | How sample size was determined. | 8 |
| Sample size | [#7b](https://www.goodreports.org/consort/info/#7b) | When applicable, explanation of any interim analyses and stopping guidelines | 8 |
| Randomization - Sequence generation | [#8a](https://www.goodreports.org/consort/info/#8a) | Method used to generate the random allocation sequence. | 7 |
| Randomization - Sequence generation | [#8b](https://www.goodreports.org/consort/info/#8b) | Type of randomization; details of any restriction (such as blocking and block size) | 7 |
| Randomization - Allocation concealment mechanism | [#9](https://www.goodreports.org/consort/info/#9) | Mechanism used to implement the random allocation sequence (such as sequentially numbered containers), describing any steps taken to conceal the sequence until interventions were assigned | 7 |
| Randomization - Implementation | [#10](https://www.goodreports.org/consort/info/#10) | Who generated the allocation sequence, who enrolled participants, and who assigned participants to interventions | 7 |
| Blinding | [#11a](https://www.goodreports.org/consort/info/#11a) | If done, who was blinded after assignment to interventions (for example, participants, care providers, those assessing outcomes) and how. | N/A |
| Blinding | [#11b](https://www.goodreports.org/consort/info/#11b) | If relevant, description of the similarity of interventions | N/A |
| Statistical methods | [#12a](https://www.goodreports.org/consort/info/#12a) | Statistical methods used to compare groups for primary and secondary outcomes | 12-13 |
| Statistical methods | [#12b](https://www.goodreports.org/consort/info/#12b) | Methods for additional analyses, such as subgroup analyses and adjusted analyses | 12-13 |
| **Results** |  |  |  |
| Participant flow diagram (strongly recommended) | [#13a](https://www.goodreports.org/consort/info/#13a) | A [diagram](https://www.goodreports.org/static/docx/diagrams/CONSORT-2010-Flow-Diagram.docx) is strongly recommended. For each group, the numbers of participants who were randomly assigned, received intended treatment, and were analysed for the primary outcome | *Figure1* |
| Participant flow | [#13b](https://www.goodreports.org/consort/info/#13b) | For each group, losses and exclusions after randomization, together with reason | *Figure1* |
| Recruitment | [#14a](https://www.goodreports.org/consort/info/#14a) | Dates defining the periods of recruitment and follow-up | N/A |
| Recruitment | [#14b](https://www.goodreports.org/consort/info/#14b) | Why the trial ended or was stopped | N/A |
| Baseline data | [#15](https://www.goodreports.org/consort/info/#15) | A table showing baseline demographic and clinical characteristics for each group | 14, Table 2 and 3 |
| Numbers analysed | [#16](https://www.goodreports.org/consort/info/#16) | For each group, number of participants (denominator) included in each analysis and whether the analysis was by original assigned groups | 14, Table 2 and 3 |
| Outcomes and estimation | [#17a](https://www.goodreports.org/consort/info/#17a) | For each primary and secondary outcome, results for each group, and the estimated effect size and its precision (such as 95% confidence interval) | 15-25 |
| Outcomes and estimation | [#17b](https://www.goodreports.org/consort/info/#17b) | For binary outcomes, presentation of both absolute and relative effect sizes is recommended | 15-25 |
| Ancillary analyses | [#18](https://www.goodreports.org/consort/info/#18) | Results of any other analyses performed, including subgroup analyses and adjusted analyses, distinguishing pre-specified from exploratory | 15-25 |
| Harms | [#19](https://www.goodreports.org/consort/info/#19) | All important harms or unintended effects in each group (For specific guidance see CONSORT for harms) | N/A |
| **Discussion** |  |  |  |
| Limitations | [#20](https://www.goodreports.org/consort/info/#20) | Trial limitations, addressing sources of potential bias, imprecision, and, if relevant, multiplicity of analyses | 29 |
| Interpretation | [#22](https://www.goodreports.org/consort/info/#22) | Interpretation consistent with results, balancing benefits and harms, and considering other relevant evidence | 26-28 |
| Registration | [#23](https://www.goodreports.org/consort/info/#23) | Registration number and name of trial registry | 8 |
| **Other Information** |  |  |  |
| Protocol | [#24](https://www.goodreports.org/consort/info/#24) | Where the full trial protocol can be accessed, if available | 7 |
| Funding | [#25](https://www.goodreports.org/consort/info/#25) | Sources of funding and other support (such as supply of drugs), role of funders | N/A |
