# Supplementary material for: The effect of comprehensive intervention for childhood obesity on dietary diversity among younger children: Evidence from a school-based randomized controlled trial in China
Source: PLoS One. 2020 Jul 17;15(7):e0235951. doi: 10.1371/journal.pone.0235951 (PMC7367455; doi:10.1371/journal.pone.0235951)
Supplement: S1 File — (DOC) [file pone.0235951.s003.doc]

# Study protocol (main points)

# The nutrition-based comprehensive intervention study on childhood obesity in China

Objective

The main purpose of the intervention study is to evaluate the feasibility and effectiveness of the comprehensive intervention program for childhood obesity which combined nutrition education and physical activity interventions. The second purpose of the intervention study is to compare the cost-effectiveness of the comprehensive intervention strategy with two other interventions, one only focuses on nutrition education, the other only focuses on physical activity. The final aim of the study is to raise the awareness of the national childhood obesity prevention and control policy recommendations

Study design

The study will be designed as a multi-centred randomiised controlled trial, which included 6 centres located in Beijing, Shanghai, Chongqing, Shandong province, Heilongjiang province and Guangdong province. Both nutrition education and physical activity intervention will be implied in all intervention schools of 5 cities, while in Beijing, nutrition education intervention will be implied in 3 schools and physical activity intervention among the other 3 schools. Nothing will be done in control schools.

Participants

A total of 9750 primary students (grade 1 to grade 5, aged 7-13 years) will participate in baseline and intervention researches of questionnaire, physical examination, physical quality test and blood biochemistry and so on. The study design in each center is presented in Figure 1. The method of two-step cluster sampling will be adopted. The first step is that randomly selected 6 schools from each centre assigned to either intervention (3 schools) or control condition（3 schools）. The second step is that randomly choose 2 classes from each grade, totally, around 250 subjects will be randomly selected from each school.

Figure 1 Study design in each center

Sample size calculations

The variable used for the calculation of sample size is the BMI changes. To detect a difference of 0.7 kg/m^2^ of BMI changes between the intervention and control groups, the minimum number required would be 3 schools in each center with 250 students in each school. The intraclass correlation is assumed to be 0.05. The sample size of 7500 students from 30 schools located in 5 centers has 90% power to detect a mean between-group difference in BMI of 0.7 units with an effect size of 0.64. Statistical significance is set at 5% (two-sided). Another 2250 students from 9 schools are randomly chosen only for cost-effect analysis, 3 schools for nutrition intervention, 3 schools for physical activity intervention and another 3 schools are treated as control schools.

Intervention

Three interventions will be included in present study: nutrition education, physical activity intervention and comprehensive intervention, the detail strategies are shown in Table 1.

**Table 1 Contents of interventions**

| Group | Interventions |
| --- | --- |
| control group | No intervention |
| Physical activity intervention group | 1. "Happy 10" campaign(2 times/day 10 minutes/time or 1 time/day 20 minutes/time)  2. Parents , health workers / health education teacher: "happy 10" training. |
| Dietary intervention group | 1. Students take part in "Nutrition and Health" classes  2. Parents participate. in "nutrition and health" classes. Give parents corresponding publicity materials.  3. Teacher, health / health education teacher participate in "nutrition and health" classes. Give them corresponding publicity materials.  4.School canteen managers, operators, competent leadership and staff room participate in "nutrition and health" classes.  5. Give them corresponding publicity materials. |
| Comprehensive (Dietary & Physical activity) intervention group | Happy 10+nutrition education  1. Students take part in "Nutrition and Health" classes  2. "Happy 10" campaign(2 times/day 10 minutes/time or 1 time/day 20 minutes/time  3. Parents participate. in "nutrition and health" classes. Give parents corresponding publicity materials.  4. Teacher, health / health education teacher participate in "nutrition and health" classes. Give them corresponding publicity materials. And give them a "happy 10" training.  5. School canteen managers, operators, competent leadership and staff room participate in "nutrition and health" classes. |

**Staff Training**

Staffs from National Institute for Nutrition and Food Safety, Chinese CDC had trained the study group members from the cooperation center for five days. Teachers, usually classroom tutors and/or health educators, will attend a two-days training session conducted by the staff of their center with the training slides and videos provided by Chinese CDC. They could learn how to integrate the program into the school curriculum, and how to perform the activities. Slides and videos about nutrition, childhood obesity, risk factors, health consequences, and prevention will be prepared by Chinese CDC and provided to school teachers. Teachers model the lessons to ensure that they understood the recommended techniques and strategies for implementation.

**Outcome measures**

Measurements will be collected in the summers of 2009 (baseline) and in summer 2010 (intervention). Children will be fasted the night before and measured the next morning by trained research staff. Consistent assessment methods will be used throughout the study.

Tables 2 Description of outcomes

| Name of Outcome | Category of Outcome | Description |
| --- | --- | --- |
| Physical examination | Primary outcome | Height, weight, waist circumference, blood pressure |
| Body Composition | Primary outcome | Bioelectrical impedance, single-standard Water Law |
| Blood biochemical indices | Primary outcome | Blood glucose, blood cholesterol, blood triglycerides, blood high-density lipoprotein, low density lipoprotein in blood, blood insulin |
| Dietary intake situations | Primary outcome | 24-hour record of 3 days dietary questionnaire |
| Physical activities | Primary outcome | Seven days of physical activity recall questionnaire, wearing the energy monitoring device, a 24-hour physical activity record sheet |
| Physical measurements | Primary outcome | Standing long jump, 50 m, 50 m × 8 from the run |
| School basic conditions | Secondary outcome | Number of students, the class distribution of the floor, the school's facilities and equipment, sports arrangements, nutrition-related staffing, students in the school dining situation |
| Obesity-related knowledge, attitudes and practices | Primary outcome | Primary school students questionnaire, parents questionnaire, teacher questionnaire, food service personnel questionnaire |
| Cost | Primary outcome | Cost related to intervention material, training, supervisor, teachers time input, transportation, |

Analysis strategy

Initially, the descriptive statistics will be calculated for all variables considered. Anthropometric measurements at baseline will be compared between intervention and control groups using multivariate regression analysis with age and sex in the model. The effects of intervention will be analyzed using mixed procedure, with BMI changes from baseline as the primary outcome variable. The fixed effects include baseline BMI, age, sex, and the intervention group. The schools within center will be treated as a random effect variable. Similar mixed models will be constructed for other outcome variables, including weight, height, BMI z score, fat free mass, fat mass, percent body fat, physical fitness, as well as the chronic disease factors including glucose, insulin, TG, TC, HDL, LDL and blood pressure.

The intervention effect on body composition will be compared according to the real diet and physical activity changes while the intervention effect on chronic disease factors will also be compared between students whose BMI z score increased or not.

Both cost-effectiveness and cost-benefit will be economically analyzed to compare the three strategies, including nutrition education intervention, physical activity intervention and the comprehensive intervention.
