# Supplementary material for: The effect of comprehensive intervention for childhood obesity on dietary diversity among younger children: Evidence from a school-based randomized controlled trial in China
Source: PLoS One. 2020 Jul 17;15(7):e0235951. doi: 10.1371/journal.pone.0235951 (PMC7367455; doi:10.1371/journal.pone.0235951)
Supplement: S2 File — (PDF) [file pone.0235951.s004.pdf]

课题任务书编号：2008BAI58B05　　　　　　　　密级：公开级

国家科技支撑计划课题任务书

（试行）

项目名称：	营养膳食对健康影响的研究
课题名称：	以膳食营养为主的儿童肥胖综合防控技术的研究
项目组织单位：	卫生部
课题承担单位：	中国疾病预防控制中心营养与食品安全所
课题负责人：	马冠生
起止年限：	2008年12月31日至2010年12月31日

中华人民共和国科学技术部

2009年05月08日

005-BA-2007-10330404-7

填 写 说 明

1. 任务书甲方为科技支撑计划项目牵头组织单位，乙方为课题承担单位。

2. 课题任务书编号由科技部统一规定。

3. 课题密级由课题承担单位提出建议，科技部项目主管司认定。

4. 课题本年度经费来源与支出预算，须与课题预算书一致。

5. 任务书签订流程:

（1）任务书由课题承担单位编写，与课题预算书一并报项目组织单位;

（2）项目组织单位汇总审定任务书，报科技部业务主管司核准;

(3)课题承担单位通过科技部门户网站(www.most.gov.cn)上的“国家科技计划项目申报中心”与“国家科技经费预算申报管理中心”进行相关内容的申报，并经申报系统软件打印书面材料(非由申报系统软件打印的书面材料，或书面材料与网上申报材料不一致的课题不予受理);

(4)任务书 A4 一式八份，由项目牵头组织单位与课题承担单位签订，项目组织单位两份；课题承担单位一份；课题承担单位主管部门和所在地方科技厅（科委）各一份；科技部三份。

国家科技支撑计划课题信息表

<table>
<tr><td colspan="2">课题编号</td><td colspan="6">2008BAI58B05</td></tr>
<tr><td colspan="2">课题名称</td><td colspan="6">以膳食营养为主的儿童肥胖综合防控技术的研究</td></tr>
<tr><td colspan="2">项目组织单位</td><td colspan="6">卫生部</td></tr>
<tr><td colspan="2">密　　级</td><td colspan="3">公开级</td><td colspan="2">参加单位总数</td><td>7个</td></tr>
<tr><td rowspan="5">课题承担单位</td><td>名　称</td><td colspan="6">中国疾病预防控制中心营养与食品安全所</td></tr>
<tr><td>单位所在地</td><td colspan="3">北京市</td><td colspan="2">组织机构代码</td><td>400019034</td></tr>
<tr><td>通讯地址</td><td colspan="3">北京市朝阳区潘家园南里7号</td><td colspan="2">邮编</td><td>100021</td></tr>
<tr><td>单位性质</td><td colspan="6">事业型研究单位</td></tr>
<tr><td colspan="2">上级行政主管部门</td><td colspan="5">卫生部</td></tr>
<tr><td rowspan="8">其他主要参加单位</td><td colspan="2">序号</td><td colspan="3">单　位　名　称</td><td colspan="2">组织机构代码</td></tr>
<tr><td colspan="2">1</td><td colspan="3">山东大学</td><td colspan="2">495570303</td></tr>
<tr><td colspan="2">2</td><td colspan="3">广州市疾病预防控制中心</td><td colspan="2">737179017</td></tr>
<tr><td colspan="2">3</td><td colspan="3">哈尔滨医科大学</td><td colspan="2">414002989</td></tr>
<tr><td colspan="2">4</td><td colspan="3">北京大学</td><td colspan="2">400002259</td></tr>
<tr><td colspan="2">5</td><td colspan="3">复旦大学</td><td colspan="2">425006117</td></tr>
<tr><td colspan="2">6</td><td colspan="3">重庆医科大学附属儿童医院</td><td colspan="2">450405742</td></tr>
<tr><td colspan="7"></td></tr>
<tr><td rowspan="6" colspan="2">课题负责人</td><td>姓　名</td><td>马冠生</td><td>性　别</td><td colspan="3">男</td></tr>
<tr><td>学　位</td><td>博士</td><td>出生日期</td><td colspan="3">1963-04-08</td></tr>
<tr><td>职　称</td><td>高级职称</td><td>专　业</td><td colspan="3">预防医学类</td></tr>
<tr><td>所在单位</td><td colspan="5">中国疾病预防控制中心营养与食品安全所</td></tr>
<tr><td>证件类型</td><td>身份证</td><td>证件号码</td><td colspan="3">310103196304081618</td></tr>
<tr><td>联系电话</td><td>67776285</td><td>E-mail</td><td colspan="3">mags@chinacdc.net.cn</td></tr>
<tr><td colspan="2">参加课题人数</td><td>63人。其中：</td><td colspan="5">高级 34 人，中级 12 人，初级 6 人，其他 11 人；</td></tr>
</table>

		博士 20 人，硕士 25 人，学士 17 人，其他 1 人。
投入人月数	778 人月（本课题满月度工作量人员数）	
起始时间	2008 年 12 月 31 日	终止时间 2010 年 12 月 31 日
课题活动类型	应用基础研究	
应用行业领域	人口与健康	
创新类型	原始创新	
主要研究内容(100 字以内)	分析膳食营养和身体活动等因素对儿童肥胖的影响，研制以膳食营养为主的儿童肥胖的综合防控技术，开展以膳食营养为主的综合干预关键技术的多中心随机对照研究，对防控技术进行评估和验证，提出儿童肥胖防控的政策建议。	
预期成果	论文和著作　研究(咨询)报告　其他	
预期知识产权	获得国外发明专利 0 项，国内发明专利 0 项，其他 0 项。	
预期技术标准制定	无	
产学研联合	是	
经费预算	366.0 万元，其中专项经费 366.0 万元。	

课题信息表填表说明

1. 带□的条目，请根据条目后所列选项，请在“□”内打✓。

2. 项目组织单位：指课题任务书的甲方单位，按公章的详细名称填写，不要填简称。代码请按所附代码表填写。

3. 课题承担单位所在地：课题承担单位，指课题任务书的乙方。所在地只填到所在省、自治区、直辖市。代码按所附代码表填写。

4. 课题承担单位性质，先按所列大类选项，代码请根据本单位的情况按所附代码表填写。

5. 课题承担单位上级行政主管部门及代码，请根据承担单位上级行政主管单位的隶属情况填写。凡隶属于地方的，填写所隶属的省、自治区、直辖市科技厅（委）代码，凡隶属于国务院部委及其直属单位的，填写国务院部委代码请按所附代码表填写。

6. 参加单位总数：包括承担单位、合作单位、协作单位在内的单位总数。

7. 课题承担单位名称：请按公章的详细名称填写。地址应详细到县（区）、街（路）门牌号。

8. 课题负责人：请按课题任务书填写。

9. 课题组人数：包括课题负责人在内的参加该课题研究工作的所有人员。

一、目标与任务

（①课题研究目标；②课题研究内容及任务分解：要解决的主要技术难点和问题，技术方案和创新点等。）

1. 课题研究目标

(1) 研制以膳食营养为主的儿童肥胖的综合防控技术；

(2) 开展以膳食营养为主的综合防控技术的多中心随机对照研究；

(3) 评估综合防控技术的成本-效益；

(4) 提出全国儿童肥胖预防控制的政策建议。

2. 课题研究内容

分析儿童肥胖的影响因素，包括饮食行为、膳食营养、身体活动等，研制以膳食营养为主的儿童肥胖的综合防控技术；开展以膳食营养为主的综合干预关键技术的多中心随机对照研究，对防控技术进行评估和验证；评估综合防控技术对儿童饮食行为、膳食营养摄入、身体活动、体成分、生理参数（血压、血糖、胰岛素、血脂相关指标等）、及肥胖率的作用；利用成本-效益分析技术对以膳食营养为主的儿童肥胖的综合防控技术进行评估，并提出防控儿童肥胖的政策建议。

3. 主要技术难点和问题分析

近年来我国儿童肥胖率迅速上升，已经成为我国的公共卫生问题。肥胖本身是一种疾病，并且是多种慢性非传染性疾病的危险因素。肥胖一旦发生，往往会持续下去，很难完全恢复正常，长期以来的经验表明成人肥胖的临床治疗是不成功的，在儿童也是这样。

儿童时期是生长发育、行为和生活方式形成的关键时期。儿童时期形成的体型和行为及生活方式，往往会伴随一生。在儿童时期，提高健康意识，培养健康的行为和生活方式，采取措施预防超重和肥胖的发生是最有效、最经济的控制整个人群肥胖及其他慢性病的关键。

由于我国儿童少年肥胖的增长尚处于快速上升的初期，因此当前正是预防和控制的紧要关头，及时抓住时机采取行之有效的措施，不仅可减缓我国儿童少年肥胖的发展趋势，还可缓解将来与肥胖相关的慢性病的增长。

儿童时期是生长发育的关键时期，既为儿童肥胖防治提供了机遇，但同时也增加了肥胖干预的难度，因为儿童需要足够的能量和营养素来满足生长发育的需要。如何合理控制儿童的饮食，既可限制过度能量和脂肪摄入，又能保证儿童少年充足的营养摄入是本研究的技术难点。

理论研究表明，控制目前中国人群的肥胖流行趋势，需要每天的能量消耗增加或能量摄入减少 45 千卡。上个世纪末的 10 年内，中国成年人平均体重从 55.4 千克增长到 59.1 千克，超重肥胖率从 9.0%增长到 23.2%，理论上讲，体重每增加 1 千克需要能量蓄积 7700 千卡，如果要控制人群中 90%的体重增长，需要平均每天少蓄积 22.5 千卡的能量，假设能量利用率为 50%，则每天能量消耗增加 45 千卡或每天能量摄入减少 45 千卡，坚持一年，则可达到控制人群超重肥胖率不再上升的趋势。分析儿童体重变化和超重肥胖率的增加趋势，得到相似的结论，即每天能量消耗增加值和能量摄入减少值相加达到 40-50 千卡，持续一年，即可达到控制儿童超重肥胖流行趋势的目的。如果能持之以恒，长此以往，理论上讲，完全可以控制儿童肥胖的流行和发展。45 千卡/天的能量意味着每天多进行 10-15 分钟中等强度身体活动或少吃 5 克烹调油。

横断面分析表明，能量摄入对能量平衡状态有很大影响，与肥胖危险性密切相关。与正常体重儿童相比，超重或肥胖的儿童膳食能量、蛋白质及脂肪摄入量高，而碳水化合物摄入量比较低；食用油及肉和奶的消费量高，而粮谷类食物和蔬菜消费量低。在控制父母体重和社

会经济学特征的相对影响后，过多的食用油消费成为影响儿童肥胖的唯一的膳食因素。中国居民膳食指南推荐的食用油脂摄入量应在2 5克以下，但70％的肥胖儿童的油脂摄入量超出了这一标准，因此适当控制儿童烹调油摄入量，将其控制在膳食指南推荐摄入量范围内，既可以达到控制儿童过度体重增长的目的，又可以保证脂肪摄入量在推荐摄入量范围内，满足儿童生长发育的需要。缺乏身体活动，久坐少动时间过长是导致肥胖高发的另一主要因素。多因素分析表明长时间看电视和玩电子游戏，或长期不参加户外身体活动，促进肥胖发生。二十世纪九十年代与八十年代相比，英国和美国儿童步行或骑车活动减少，而坐车上学的比例增加。我们对2002年中国居民营养与健康状况调查资料的分析结果也发现，超重肥胖儿童平均每天比正常体重儿童中/高等强度身体活动少30分钟。

干预研究进一步证实了每天增加十分钟中/高等身体活动，可有效促进儿童的身高发育，控制儿童体重的过渡增长。北京市小学生“快乐十分钟”研究结果表明，通过每天的“快乐十分钟”活动，儿童的能量消耗平均每天增加25-35千卡/天，与对照组相比，平均身高每年多增长0.24-0.35厘米（1992-2002年10年间我国儿童身高平均增长了3.3厘米），同时体重增加幅度减少0.2-2.3千克，在对照组超重肥胖率继续上升的同时，干预组儿童超重肥胖率有所下降。目前关于膳食干预对儿童肥胖干预效果的研究国内尚未见报道。

针对儿童超重肥胖防治的技术难点和以往的研究的结果，本研究将利用全国性的资料进一步分析影响儿童肥胖及身高发育的关键因素，在充分分析不同调查点儿童饮食特点的基础上，根据不同食物和营养素的特点，结合儿童生长发育的需要，制定适合个体及群体的肥胖干预措施，平衡膳食的推广和烹调油的控制将作为膳食干预的两个切入点，引导儿童形成健康的饮食方式，在保证儿童健康生长发育

的基础上，适宜控制体重增长。并对不同干预措施进行成本-效益分析，找到最经济有效的措施，以供进一步推广应用。

4. 技术方案和创新点

技术路线图：

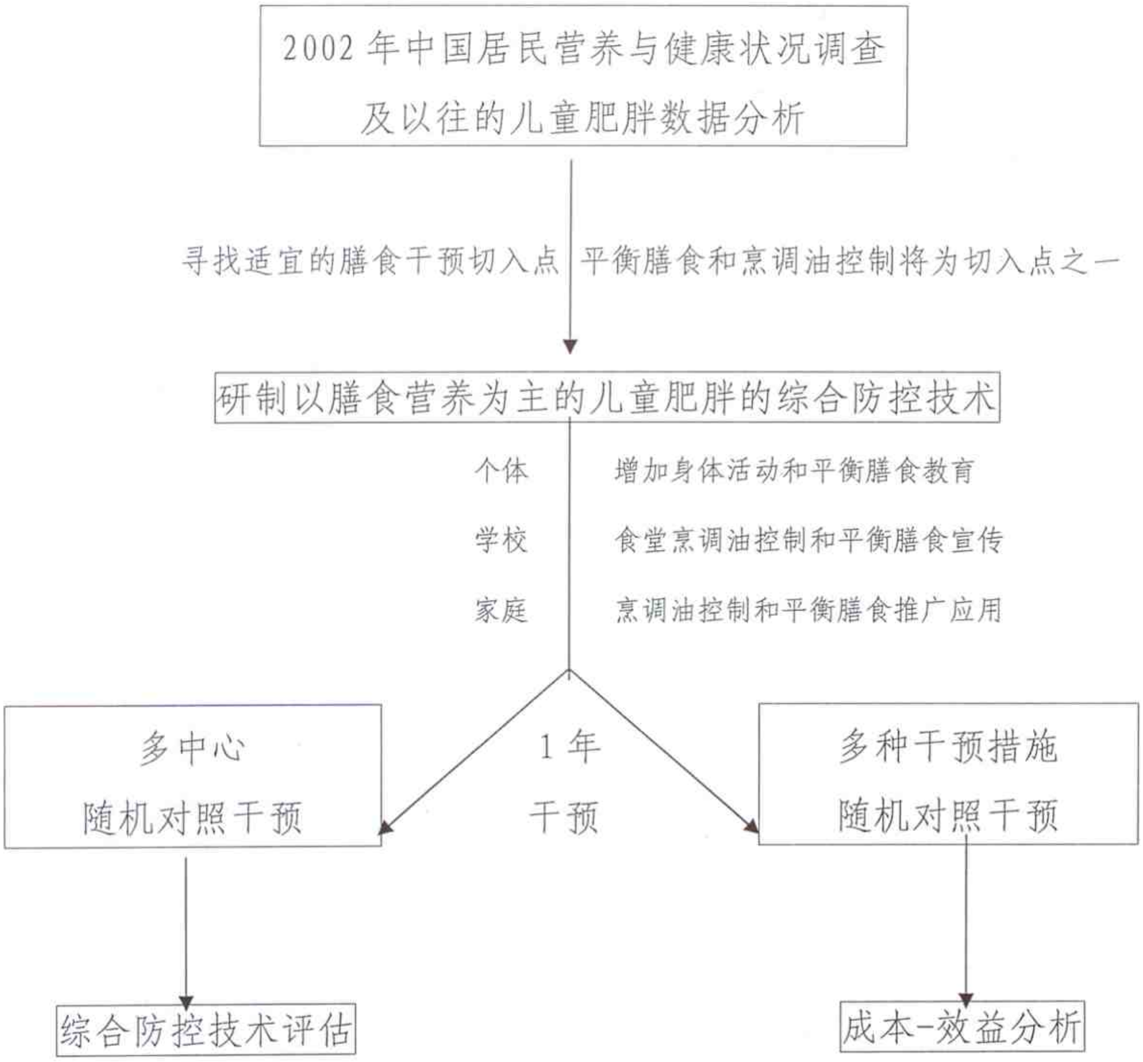

研究设计：多中心随机对照干预试验，本研究是一项在学校环境里进行随机对照干预试验。因为个体化的随机分配是无法实现的，因此参与的学校被分为干预校和对照校，七个中心，每个中心选择1个干预学校和1个对照学校。

研究对象：小学生，计算样本量每个调查点约为1500名学生。

纳入标准：学校统一供餐。

排除标准：拟作为研究对象的学生如果患有严重疾病，比如先天性心脏病，不能承受重身体活动和膳食控制的，需要从研究中排除。

持续时间：本项目将持续两年，其中干预时间为1学年。

数据收集：在干预前后各进行一次。测定小学生的晨起空腹体重，身高、体成分，血压，抽取静脉血测定血糖、胰岛素、血脂等相关指标。

利用问卷调查的方式调查学生膳食、身体活动、家庭基本信息等。

数据分析：（1）首先利用2002年中国居民营养与健康状况调查数据分析儿童肥胖的影响因素，寻找适宜的儿童肥胖干预切入点；（2）结合以往研究结果制定儿童肥胖的综合防控技术；（3）分析干预组和对照组小学生体格发育、身体成分和血生化指标在干预前后变化的差异性；（4）利用成本-效益分析技术对以膳食营养为主的儿童肥胖的综合防控技术进行评估。

创新点：首次在全国开展多中心的儿童肥胖的综合防控技术推广和效果评估，将科研与推广相结合，兼备学术意义和现实收益；首次进行不同干预措施的成本-效益分析研究，结果将为国家制定全国的干预策略提供科学的依据。

二、预期成果及考核指标

（①主要技术指标：如形成的知识产权、技术标准、新技术、新产品、新装置、论文专著等数量、指标及其水平等；②主要经济指标：如技术及产品应用所形成的市场规模、效益等；③项目实施中形成的示范基地、中试线、生产线及其规模等；④人才队伍建设；⑤其他应考核的指标。）

1. 主要技术指标

主要技术指标（如形成的知识产权、技术标准、新技术、新产品、新装置、论文专著等数量、指标及其水平，与国内外同类技术或产品的竞争分析，满足项目所依托的重大工程建设或重大装备研制的需求情况等）

新技术：儿童肥胖综合防控技术

论文专著：国际同行评议英文期刊将发表论文至少 2 篇：（1）综合防控技术的评估；（2）不同干预措施的成本-效益分析。国家级专业学术期刊发表论文 3-5 篇。

政策建议报告：根据研究结果，将提供关于我国儿童肥胖干预的政策研究建议报告。

完善《中国学龄儿童少年超重和肥胖预防与控制指南》（试用）版：根据研究结果，进一步完善指南，争取正式出版发行，在全国范围推广应用。

2. 主要经济、社会、环境效益

通过儿童肥胖综合防控技术的推广，预计儿童平均身高可每年多增长 0.2 厘米，而体重保持不变，体脂蓄积减缓。诺贝尔奖获得者英国经济学家 Fogel 教授研究认为，经济效益的增加取决于国民身体素质和营养状况的加强。如果我们通过适宜干预，增强儿童体质，减缓甚至控制儿童肥胖的流行，将提升我国国民的综合竞争力，其经济效益是不容忽视的。

儿童肥胖不仅影响到儿童体型和自信心，原来在成年期才出现的糖尿病和心血管疾病已经在儿童期开始积聚。另外，儿童少年肥胖最严重的后果是肥胖向成年期的延续，包括肥胖体型的延续、引起肥胖的生活方式的延续，而延续到成年的肥胖又导致相关的成人心脑血管疾病、2型糖尿病等慢性非传染性疾病发病的危险增加，严重威胁人群的身体素质和健康水平，给社会经济发展、民族素质的提高带来了巨大负担。

研究表明，2003 年中国超重和肥胖所造成的高血压、糖尿病、冠心病、脑卒中的直接经济负担分别为 89.7,25.5,22.6 和 73.3 亿元，4 种病合计归因于超重和肥胖的直接经济负担高达 211.1 亿元人民币，占 4 种病合计直接疾病负担的 25.5%。如果不采取相应措施，当超重与肥胖率比值达到 1.1:1 时，归因于超重和肥胖导致 4 种病的直接经济负担将达到 369.9 亿元。如果我们通过及时有效的干预措施，适当延缓控制儿童肥胖的发生发展，其经济效益也不容忽视。

3. 课题实施中可能形成的示范基地、中试线、生产线及其规模

在六个合作中心形成儿童肥胖防治示范基地，在干预有效的基础上，进一步推广到其他学校和地区。

4. 人才队伍建设

（1）在全国带动培养一批中青年儿童肥胖专家，进而带动全国范围内的儿童肥胖防治工作。

（2）学校卫生队伍培训：培训干预学校卫生老师及学校领导，进行儿童肥胖综合干预，以及营养、身体活动、儿童肥胖及其健康危害的知识技能培训；

（3）学生：学生通过综合防控技术的应用，在知、信、行方面都得到培训，尤其是对于那些对肥胖比较关注，控制体重的

主观能动性较强的学生，其行为改善将受益终生。

（4）学校：学校有能力自行制定以学校为基地的肥胖预防计划。

（5）硕士博士培养：在项目进行过程中，将培养硕士 7-8 名、博士 2-3 名；

（6）在培训活动和项目研究中，将改进和检验各种培训教材，并且会得到很多有价值的经验，可以在将来由教育部门提供给其他学校，供全国中小学生使用。

5. 其它应考核的指标

其它考核指标包括各调查点实际投入的人力、物力，在干预过程中遇到的问题和解决措施等与儿童肥胖干预有关的信息。

三、课题年度计划及年度目标

1. 年度计划

序号	进度安排<br>任　务	第一年<br>（2008.12）		第二年<br>(2009.1-12)		第三年<br>(2010.1-12)	
		上半年	下半年	上半年	下半年	上半年	下半年
1	项目启动会						
2	文献收集和资料分析		*	*			
3	个体和综合防控技术的制定			*			
4	基线资料收集			*			
5	综合防控技术的实施				*	*	
6	终期资料收集					*	
7	资料录入清理				*	*	*
8	资料分析报告						*
9	论文撰写						*

2. 课题各年度目标及考核指标

第一年：项目启动，各中心明确任务和分工；开始相关文献收集和已有资料的分析

考核指标：启动会召开资料及详细分工安排记录

第二年：年度目标：综合防控技术制定及实施

考核指标：（1）综合防控技术；

（2）基线资料收集完成；

（3）综合防控技术开始实施

第三年：年度目标：综合防控评估

考核指标：（1）综合防控效果评估结果；

（2）提交综合防控技术；

（3）成本-效益分析结果；

（4）论文投稿（英文2篇，中文3-5篇）；

（5）政策建议报告提交；

（6）完善出版《中国学龄儿童少年超重和肥胖预防与控制指南》

四、课题经费来源与支出预算

单位：万元（保留两位小数）

序号	预算科目名称	合计	专项经费	自筹经费
1	一、经费支出	366.00	366.00	0.00
2	1. 设备费	19.00	19.00	0.00
3	（1）购置设备费	19.00	19.00	0.00
4	（2）试制设备费	0.00	0.00	0.00
5	（3）设备改造与租赁费	0.00	0.00	0.00
6	2. 材料费	117.60	117.60	0.00
7	3. 测试化验加工费	143.40	143.40	0.00
8	4. 燃料动力费	0.00	0.00	0.00
9	5. 差旅费	16.00	16.00	0.00
10	6. 会议费	5.00	5.00	0.00
11	7. 国际合作与交流费	16.00	16.00	0.00
12	8. 出版/文献/信息传播/知识产权事务费	8.00	8.00	0.00
13	9. 劳务费	16.00	16.00	0.00
14	10. 专家咨询费	4.00	4.00	0.00
15	11. 管理费	21.00	21.00	0.00
16	12.	0.00	0.00	0.00
17	13.	0.00	0.00	0.00
18	二、经费来源	366.00	366.00	0.00
19	1. 申请从专项经费获得的资助	366.00	366.00	/
20	2. 自筹经费来源	0.00	/	0.00
21	（1）其他财政拨款	0.00	/	0.00
22	（2）单位自有货币资金	0.00	/	0.00
23	（3）其他资金	0.00	/	0.00

五、课题的承担单位、参加单位及主要研究人员

课题承担单位:

中国疾病预防控制中心营养与食品安全所

主要参加单位:

山东大学、广州市疾病预防控制中心、哈尔滨医科大学、北京大学、复旦大学、重庆医科大学附属儿童医院

课题负责人

序号	姓名	性别	出生日期	证件类型	证件号码	职务	职称	专业	为本课题工作时间（%）	所在单位
1	马冠生	男	1963-04-08	身份证	310103196304081618	副所长	高级职称	预防医学类	60	中国疾病预防控制中心营养与食品安全所

主要研究人员

序号	姓名	性别	出生日期	证件类型	证件号码	职务	职称	专业	为本课题工作时间（%）	所在单位
1	胡小琪	女	1960-06-08	身份证	110104196006080085	学生营养室主任	高级职称	临床医学与医学技术类	70	中国疾病预防控制中心营养与食品安全所
2	杜琳	女	1956-04-05	身份证	440105195604052429	书记	高级职称	预防医学类	30	广州市疾病预防控制中心
3	徐贵法	男	1948-09-24	身份证	370102194809240819	教授	高级职称	预防医学类	60	山东大学公共卫生学院
4	李颖	女	1970-06-18	身份证	230106197006182027	副主任	高级职称	预防医学类	60	哈尔滨医科大学公共卫生学院
5	马军	男	1961-06-06	身份证	110108196106069719	所长/教授	高级职称	预防医学类	50	北京大学儿童青少年卫生研究所
6	郭红卫	女	1953-11-16	身份证	310101195311160069	教授	高级职称	预防医学类	40	复旦大学公共卫生学院
7	李廷玉	女	1956-01-19	身份证	510202195601191823	院长	高级职称	预防医学类	20	重庆医科大学附属儿童医院
8	李艳平	女	1972-01-22	身份证	130604197201220923	副研究员	高级职称	预防医学类	40	中国疾病预防控制中心营养与食品卫生研究所
9	刘爱玲	女	1972-11	身份	3706231972	副研	高级	预防医	80	中国疾病预防控

			-17	证	11173220	究员	职称	学类		制中心营养与食品安全所
10	王京钟	男	1963-07-24	身份证	110108630724233	研究员	高级职称	预防医学类	80	中国疾病预防控制中心营养与食品安全所
11	张倩	女	1972-08-23	身份证	140103197208233928	副研究员	高级职称	预防医学类	60	中国疾病预防控制中心营养与食品安全所
12	杜松明	女	1970-03-15	身份证	41042219700315002x	副教授	高级职称	预防医学类	90	中国疾病预防控制中心营养与食品安全所
13	郝利楠	女	1977-09-18	身份证	140103197709183404	讲师	中级职称	临床医学与医学技术类	100	中国疾病预防控制中心营养与食品安全所
14	房红芸	女	1982-02-05	身份证	370830198202050025	博士生	其他人员	预防医学类	90	中国疾病预防控制中心营养与食品安全所
15	段一凡	女	1984-07-27	身份证	110108198407274942	硕士生	其他人员	预防医学类	90	中国疾病预防控制中心营养与食品安全所
16	赵静	女	1983-07-12	身份证	130203198307122420	硕士生	其他人员	护理学类	80	中国疾病预防控制中心营养与食品安全所
17	王翠霞	女	1983-01-29	身份证	371327198301291241	硕士生	其他人员	预防医学类	80	中国疾病预防控制中心营养与食品安全所
18	檀倩影	女	1985-01-04	身份证	45072119850104394x	硕士生	其他人员	预防医学类	80	中国疾病预防控制中心营养与食品安全所
19	刘颖	女	1982-11-24	身份证	370683198211249281	硕士生	其他人员	预防医学类	40	中国疾病预防控制中心营养与食品安全所
20	张媛	女	1973-06-16	身份证	210402730616094	博士后	中级职称	预防医学类	80	中国疾病预防控制中心营养与食品安全所
21	蔺新英	女	1957-08-09	身份证	37010219570809290X	教授	高级职称	预防医学类	50	山东大学公共卫生学院
22	李军	女	1956-10-13	身份证	370102195610130641	教授	高级职称	预防医学类	50	山东大学公共卫生学院
23	赵长峰	男	1960-08-12	身份证	370102196008120816	副教授	高级职称	预防医学类	40	山东大学公共卫生学院

24	付茂笋	男	1961-01-06	身份证	370102196101060811	副教授	高级职称	预防医学类	30	山东大学公共卫生学院
25	邵丽华	女	1958-01-05	身份证	34210219580105002X	教授	高级职称	临床医学与医学技术类	30	山东大学公共卫生学院
26	崔晞	男	1955-03-01	身份证	370102195503012156	教授	高级职称	临床医学与医学技术类	30	山东大学公共卫生学院
27	刘萍	女	1964-01-05	身份证	370102196401050842	副教授	高级职称	临床医学与医学技术类	30	山东大学公共卫生学院
28	刘伟佳	男	1968-08-15	身份证	440102196808153219	副科长/副主任医师	高级职称	预防医学类	30	广州市疾病预防控制中心
29	吴家刚	男	1974-06-08	身份证	422722197406080310	医师	初级职称	预防医学类	30	广州市疾病预防控制中心
30	肖新才	男	1972-10-10	身份证	362101197210100617	副主任医师	高级职称	预防医学类	60	广州市疾病预防控制中心
31	林蓉	女	1981-12-22	身份证	440583198112222844	医师	初级职称	预防医学类	60	广州市疾病预防控制中心
32	林琳	女	1968-04-14	身份证	44010219680414442x	主管医师	中级职称	预防医学类	80	广州市疾病预防控制中心
33	张维蔚	女	1980-11-26	身份证	440402198011269025	医师	初级职称	预防医学类	80	广州市疾病预防控制中心
34	王舒然	男	1968-05-17	身份证	230103196805171312	副主任	高级职称	临床医学与医学技术类	40	哈尔滨医科大学公共卫生学院
35	张慧颖	女	1967-02-05	身份证	230103196702054842	医师	初级职称	预防医学类	60	哈尔滨医科大学公共卫生学院
36	王朝旭	男	1956-01-28	身份证	230103195601281435	研究员	高级职称	预防医学类	50	哈尔滨医科大学公共卫生学院
37	闻颖	女	1966-07-20	身份证	230103196607204822	副教授	高级职称	预防医学类	40	哈尔滨医科大学公共卫生学院
38	夏薇	女	1971-10-19	身份证	230103197110190028	副教授	高级职称	预防医学类	80	哈尔滨医科大学公共卫生学院

39	潘洪志	男	1969-12-03	身份证	230103196912031315	副教授	高级职称	预防医学类	50	哈尔滨医科大学公共卫生学院
40	赵丹	女	1977-10-31	身份证	230803197710310028	讲师	中级职称	预防医学类	60	哈尔滨医科大学公共卫生学院
41	王海俊	女	1973-10-28	身份证	450204197310281023	讲师	中级职称	预防医学类	60	北京大学儿童青少年卫生研究所
42	李榴柏	女	1966-04-17	身份证	110108196604172321	副研究员	高级职称	预防医学类	60	北京大学儿童青少年卫生研究所
43	宋逸	女	1976-01-28	身份证	110105197601289028	讲师	中级职称	预防医学类	70	北京大学儿童青少年卫生研究所
44	陈天娇	女	1977-12-06	身份证	110108197712069764	讲师	中级职称	预防医学类	70	北京大学儿童青少年卫生研究所
45	王莹	女	1956-11-28	身份证	110108195611288926	主管技师	中级职称	临床医学与医学技术类	70	北京大学儿童青少年卫生研究所
46	李珊珊	女	1983-06-01	身份证	110108198306011828	硕士生	其他人员	预防医学类	80	北京大学儿童青少年卫生研究所
47	张伋	女	1985-03-28	身份证	110108198503284921	硕士生	其他人员	预防医学类	60	北京大学儿童青少年卫生研究所
48	裴正存	男	1984-09-22	身份证	211121198409223810	硕士生	其他人员	预防医学类	60	北京大学儿童青少年卫生研究所
49	吴双胜	男	1985-06-06	身份证	320623198506065636	硕士	其他人员	预防医学类	80	北京大学儿童青少年卫生研究所
50	李百惠	女	1985-02-19	身份证	370181198502194428	硕士生	其他人员	预防医学类	60	北京大学儿童青少年卫生研究所
51	何更生	女	1965-12-19	身份证	310104196512192829	副教授	高级职称	预防医学类	20	复旦大学公共卫生学院
52	罗飞宏	男	1966-09-13	身份证	310104196609132830	副主任医师	高级职称	临床医学与医学技术类	30	复旦大学附属儿科医院
53	王劲	男	1970-01-22	身份证	360102197001225810	讲师	中级职称	预防医学类	50	复旦大学公共卫生学院
54	薛琨	女	1977-10-13	身份证	120105197710130325	讲师	中级职称	预防医学类	40	复旦大学公共卫生学院
55	陈凤麟	男	1951-12-31	身份证	310105195112312010	副主任技师	初级职称	预防医学类	50	复旦大学公共卫生学院
56	刘友学	男	1965-03-20	身份证	510212650320445	副处长	高级职称	临床医学与医	30	重庆医科大学附属儿童医院

								学技术类		
57	熊　丰	女	1958-08-16	身份证	510202195808161824	主任	高级职称	临床医学与医学技术类	30	重庆医科大学附属儿童医院
58	赵　勇	男	1976-06-20	身份证	5102251976 0620077X	讲师	中级职称	预防医学类	60	重庆医科大学附属儿童医院
59	程　茜	女	1962-07-27	身份证	5102021962 0727182X	主任	高级职称	临床医学与医学技术类	30	重庆医科大学附属儿童医院
60	朱　岷	女	1963-03-23	身份证	510202196303231844	副主任	高级职称	临床医学与医学技术类	35	重庆医科大学附属儿童医院
61	魏小平	男	1981-08-25	身份证	510781198108258170	实习研究员	初级职称	预防医学类	70	重庆医科大学附属儿童医院
62	练雪梅	女	1972-07-09	身份证	512221197207090020	讲师	中级职称	预防医学类	40	重庆医科大学附属儿童医院

六、任务书签订各方签章

项目组织单位（甲方）：

负责人（签字）：何维　　　　　　　　　　　　　　（公　章）

2009年 9 月 8 日

课题承担单位（乙方）：

课题负责人（签字）：[illegible]　　　　　　　　　　（公　章）

2009年 7 月 31 日

资金等匹配条件落实保证方

乙方主管部门或地方科技厅（科委）：　　　　　　　（公　章）

负责人(签字)：[illegible]

2009年 8 月 [illegible] 日

科技部核准意见

科技部项目主管司：　　　　　　　　　　　　　　（公　章）

负责人（签字）：杨哲之印

2009年 10月 14 日

七、共同条款

任务各方共同遵守《国家科技支撑计划管理暂行办法》(以下简称《办法》):

1. 乙方必须按要求编报年度计划执行情况、下一年度经费预算和有关统计报表，交甲方汇总后，及时上报科技部，逾期不报，科技部有权暂停拨款。

2. 任务执行过程中，乙方如需调整任务，应根据《办法》中有关规定，向甲方提出变更内容及其理由的申请报告，经甲方审核后报科技部审定后实施。未经接到正式批准书以前，双方须按原任务书履行，否则后果由自行调整的一方负责。

3. 乙方因某种原因（如：与可行性研究内容有出入、挪用经费、技术措施或某些条件不落实）致使计划无法执行，而要求中止任务，应视不同情况，部分、全部退还所拨经费；如乙方没有提出中止任务的要求，甲方可根据调查情况有权提出中止任务的处理建议，报科技部审核批准后执行。

4. 乙方承担任务所需国拨经费按《国家科技支撑计划专项经费管理暂行办法》管理和使用。

5. 甲方根据国家科技支撑计划经费开支的规定，监督经费的使用情况。凡不符合规定的开支，甲方负责提出调整意见。必要时，科技部有权直接提出调整或撤销意见。

6. 任务执行过程中，甲方无故中止任务时，所拨经费、物资不得追回，并承担善后处理所发生的费用。甲方提出变更任务书有关内容时，要与乙方协商达成书面协议，并报科技部备案后实行。

7. 若课题承担单位的上级主管部门或所在地的地方科技厅（科委），承诺课题实施需要的配套资金等条件，须在课题任务书配套条件落实保证方栏加盖公章。

8. 本任务书签订各方均负有相应的责任。若有争议或纠纷时，按《办法》有关条款处理。

9. 任务书正式文本一式八份，甲方两份、乙方一份、乙方主管部门和所在地方科技厅（科委）各一份、科技部三份。

10. 本任务书所协议的其他条款如下:
