# Supplementary material for: The effect of comprehensive intervention for childhood obesity on dietary diversity among younger children: Evidence from a school-based randomized controlled trial in China
Source: PLoS One. 2020 Jul 17;15(7):e0235951. doi: 10.1371/journal.pone.0235951 (PMC7367455; doi:10.1371/journal.pone.0235951)
Supplement: Attachment — Submitted filename: Response to Reviewers.docx [file pone.0235951.s005.docx]

30 December 2019

Dear Dr. Mahdieh, Editors and Reviewers:

We very much appreciate your thoughtful comments on our manuscript (PONE-D-19-24892, “Childhood obesity interventions only focusing on physical activity decreases dietary diversity among younger children: Evidence from a school-based randomized controlled trial in China”). We have carefully considered each comment and responded point-by-point below. We have made corresponding changes in the manuscript with tracked changes highlighting both additions and deletions.

In addition, we have carefully checked the format requirement and revised the manuscript according to the requirement of *PLOS ONE*.

We believe that your comments and our responses to them have improved our manuscript considerably, and we hope you find our revised manuscript suitable for publication.

Sincerely,

Guansheng Ma Prof.

Department of Nutrition and Food Hygiene,

School of Public Health, Peking University,

38 Xue Yuan Road,

Beijing, China.

Email: [mags@bjmu.edu.cn](mailto:mags@bjmu.edu.cn)

<Tel:13801080336>,

Fax: +86 10 82105184.

Guansheng Ma Prof.

Department of Nutrition and Food Hygiene,

School of Public Health, Peking University,

38 Xue Yuan Road,

Beijing, China.

Email: [mags@bjmu.edu.cn](mailto:mags@bjmu.edu.cn)

<Tel:13801080336>,

Fax: +86 10 82105184.

Response to Journal Requirements:

1. When submitting your revision, we need you to address these additional requirements. Please ensure that your manuscript meets PLOS ONE's style requirements, including those for file naming. The PLOS ONE style templates can be found at http://www.journals.plos.org/plosone/s/file?id=wjVg/PLOSOne_formatting_sample_main_body.pdf and http://www.journals.plos.org/plosone/s/file?id=ba62/PLOSOne_formatting_sample_title_authors_affiliations.pdf

Response: We have revised the format according to PLOS ONE’s style.

2. Please include captions for your Supporting Information files at the end of your manuscript, and update any in-text citations to match accordingly. Please see our Supporting Information guidelines for more information: <http://journals.plos.org/plosone/s/supporting-information>.

Response: We provide one supporting information file “Checklist”, and give the caption at the end of the manuscript as:

*“Supporting information*

*S1 File. S1 CONSORT Checklist”* (*Page 35, Line 656-657)*

3. We noticed you have some minor occurrence(s) of overlapping text with the following previous publication(s), which needs to be addressed:
https://doi.org/10.1186/s12991-017-0162-2
https://doi.org/10.6133/apjcn.112016.05
https://doi.org/10.1371/journal.pone.0043183
https://doi.org/10.1186/s12937-017-0299-5
https://doi.org/10.1186/s12889-016-3878-z
<http://dx.doi.org/10.5888/pcd13.160032>

Response: we have revised the overlapping text with the reference as requirements.

In your revision ensure you cite all your sources (including your own works), and quote or rephrase any duplicated text outside the Methods section. Further consideration is dependent on these concerns being addressed.

Response: we have deleted the duplicated text and made the new description, such as *“In addition to a positive relationship between DDS and nutrient intake being reported, the inverse association between DDS and chronic diseases [9-11] have been revealed by several studies.” (Page 5, Lines 95-97), and “In PA intervention schools, the “Happy 10”, which was a classroom-based physical activity program for primary school students was used [18, 24].” (Page 8, Lines 163-165), and “The 28 food groups were categorized as follows: Wheat; Fried wheat; Rice; Refined grains; Other cereals; Starch tubers; Beans; Soybean products; Nuts; Deep-color vegetables (carotene content ≥ 500 μg/100 g); Light-color vegetables (carotene content < 500 μg/100 g); Pickled vegetables; Edible fungi and algae; Fruits; Pork; Poultry; Beef/lamb/other red meat; Seafood; Organ meat; Processed meat; Eggs; Milk powder and cheese; Milk and yogurt; Catsup; Beverages; Sugar; Fast food; and Cakes [38]” (Page 11-12, Line 214-220); and “**The qualitative research data showed that low parental response could aggravate their children’s unhealthy eating behavior [51-54]. Therefore, both of them were covered in our program.” (Page 27-28, Line 396-398);*

Responses to Reviewer #1: Childhood obesity interventions only focusing on physical activity decreases dietary diversity among younger children: Evidence from a school-based randomized controlled trial in China
1.1 The title of study highlighted that focusing on physical activity decrease dietary diversity among children, while the evidences provided in the introduction, method, result and discussion weren’t enough to convince the readers. I am interested to know how Pa interventions decrease DDS but unfortunately couldn’t find strong procedure to prove it.

Response: According to the suggestions given by the reviewer as well as other reviewers, we have revised the title to reflect the overall study design, instead of only part of the intervention strategies. We have also reorganized the results accordingly. The title was revised as: *“**The effect of comprehensive intervention for childhood obesity on dietary diversity among younger children: Evidence from a school-based randomized controlled trial in China”* (*Page1, Line4-6*)

*“*Method*: Three intervention groups included “Nutrition education intervention (NE), physical activity intervention (PA) and comprehensive intervention including both NE and PA (CNP) were carried out separately for 2 semesters.”* (*Page3, Line57-59*)

The results and conclusions in abstract were revised as:

*“****Results***

*The intervention effects per day of comprehensive intervention group were 0.01 (95% CI: -0.05, 0.08; p = 0.731) on DDS9, 0.04 (95% CI: -0.08, 0.16; p = 0.521) on DDS28 and 0.08 (95% CI: -0.1, 0.26; p = 0.377) on FVS of overall diet, which was 0.09 (95% CI: 0.05, 0.13; p < 0.001) on DDS9, 0.05 (95% CI: 0, 0.11; p = 0.049) on DDS28 and 0.07 (95% CI: 0.01, 0.12; p = 0.024) on FVS of dietary scores of breakfast only. Additionally, CNP group had greater increases in cereals, meat and fruits, and more decreases in eggs, fish and dried legumes consumption proportions as compared with the control group. Decreasing side effect on dietary diversity and food variety were found for PA intervention, but not for NE intervention only.*

***Conclusions***

*Though the comprehensive obesity intervention didn’t improve the overall dietary diversity per day, the positive intervention effects were observed on breakfast foods and some foods’ consumption.”* (*Page3-4, Line 63-77*)

The results and conclusions in main text were revised as:

*“**Dietary diversity and food variety*

*The intervention effects on DDS and FVS according to dietary intake are shown in Table 4 for CNP study and in Table 5 for NE or PA study. Compared with the control group, the effects of CNP were 0.01 (95% CI: -0.05, 0.08; p = 0.731) on DDS9, 0.04 (95% CI: -0.08, 0.16; p = 0.521) on DDS28 and 0.08 (95% CI: -0.1, 0.26; p = 0.377) on FVS. No significant difference of intervention effects was found across subgroups.* *When we evaluated the dietary scores based on the breakfast foods only, significant intervention effects of* *CNP group were observed on DDS9 (0.09; 95% CI: 0.05, 0.13; p < 0.001), DDS28 (0.05; 95% CI: 0, 0.11; p = 0.049) and FVS (0.07; 95% CI: 0.01, 0.12; p = 0.024).”*(Page15-16, Line 286-295)

*“The effects on DDS9, DDS28 and FVS were 0.06 (95% CI: -0.14,0.26; p =0.567), 0.02 (95% CI: -0.33,0.38; p =0.893) and 0.23 (95% CI: -0.29,0.76; p =0.386) in the NE group and -0.27 (95% CI: -0.47,-0.06; p =0.01), -0.55 (95% CI: -0.91,-0.19; p =0.003) and -0.74 (95% CI: -1.27,-0.22; p =0.006) in the PA group, respectively. No significant effects on DDS or FVS were found at breakfast in NE group or PA group”*

(*Page22, Line 313-317*)

*“****Conclusions***

*These results indicated that though CNP had no significant effect on overall dietary diversity and food variety per day,* *the significant effects were shown on some foods’ consumption, and the dietary diversity and food variety increases appeared at breakfast significantly. Children exposed only to the physical activity intervention or nutrition education intervention as an obesity intervention didn’t appear the positive effect on dietary diversity either. We should pursue a comprehensive intervention approach to changing school policies and practices that addresses both nutrition education and physical activity over time.”* (*Page 29-30, Line 435-442*)

1.2 Method
The intervention elements are unclear for all comprehensive, nutrition education, and PA. Please, clarify the interventions program in details.

Response: we have supplied some detailed description of three intervention measures in this program in method.

*“****Intervention implementation***

*Table 1 shows the intervention measures for different intervention groups. In NE intervention schools, one nutrition education handbook was developed [23] for the students, courses on nutrition and health for the students, parents, teachers and health workers were designed separately. Furthermore, “Dietary Pagoda for Chinese people” posters were displayed in the classrooms for NE intervention groups.* *In PA intervention schools, the “Happy 10”, which was a classroom-based physical activity program for primary school students was used [18, 24]. Furthermore, students, parents, health workers and teachers received the PA education from the program. To improve the home environment, the parents in the intervention groups were also involved, including but not limited to sending them physical activity education bulletins. In CNP schools, all above interventions including both NE and PA were implemented.* *In order to ensure the normal operation of the intervention, the supervisors from project office went to each center to carry out project supervision during the program period. The process evaluation indicated that the school carried out as proposal.**”* (*Page 8-9, Line 158-172*)

*“****Table 1.*** ***The Interventions for Different Intervention Groups****.*

| ***Intervention groups*** | ***Interventions*** | ***Sites*** |
| --- | --- | --- |
| *Nutrition education (NE)* | *1. The nutrition handbook was distributed to students.*  *2. Courses on nutrition and health were given 6 times to students, 2 times to parents and 4 times to teachers and health workers. The content included proportion of three meals, how to choose healthy food, reducing eating out, unhealthy fast food sugar sweetened beverage and snacks.*  *3. Displaying poster of “Dietary Pagoda for Chinese people”.*  *4.2 class meetings for nutrition and health.* | *Beijing* |
| *Physical activity (PA)* | *1. Course on physical activity given to parents*  *2. Twenty-minute “Happy 10” physical activity for students on each school day*  *3. 2 class meetings for physical activity and health.* | *Beijing* |
| *Comprehensive intervention including nutrition education and physical activity (CNP)* | *All the above interventions including both NE and PA were implemented.* | *The other 5 cities*  *(Jinan, Chongqing, Harbin,*  *Guangzhou and Shanghai)* |

*”* (*Page 9-10, Table 1, Line 178-179*)

1.3 PA interventions exist but I can’t find any information about PA measurements in method and results.
Response: we have added the evaluation information about physical activity level in the methods. *“In CNP schools, all above interventions including NE and PA were implemented. In order to ensure the normal operation of the intervention, the supervisors from project office went to each center to carry out project supervision during the program period. The process evaluation indicated that the school carried out as proposal. The physical activity level was measured with energy monitor among 868 students ( 304 in CNP group and 301 in control group in 5 cities, 111 in PA group and 112 in NE group in Beijing).The energy expenditure increased higher in CNP group* *(77.0 kcal/day) than in control group (63.5 kcal/day, p = 0.967) in five cities study, higher in PA group (96.4 kcal/day) than in NE group (76.7 kcal/day, p = 0.408) in Beijing study. ”* (*Page9, Line 168-177*)

Responses to Reviewer #2: This paper reported a secondary analysis of the multicentre cluster-randomized controlled trial conducted in China between 2009 and 2010 that aimed to evaluate the effects of a comprehensive school-based intervention on childhood obesity prevention. The main trial was designed into two separate studies, conducted in Beijing and other 5 cities separately. The Beijing study involved 8 schools with 3 schools randomly allocated to receive nutrition education (NE) only, 3 school to receive physical activity (PA) only, and 2 control schools with no intervention. The other study recruited 6 schools in each of the 5 cities (30 schools in total), with 3 schools randomly allocated to receive a comprehensive intervention including both NE and PA (CNP), and 3 control schools with no intervention. The programme was implemented for 2 semesters over one year, and data were collected at baseline and at the end of the study. Among the 38 primary schools included in the trial, two classes were selected from each grade (1-5) per school, but only grades 2-4 were designed to collect the dietary records using 24-h dietary recall for 3 consecutive days. The current study aimed to explore the effects of NE and PA on dietary diversity compared to the control groups, measured using dietary diversity scores (DDS9 and DDS28) and food variety score (FVS). The proportions of different foods consumed were also calculated.

We appreciate the reviewer’s summaries.

2.1 The manuscript was well written, and the study design was clearly documented. My major comments are on the statistical analysis and results reported in this paper, which should follow the CONSORT 2010 guidelines with extension to cluster randomised trials.
Response: We appreciate the positive comments. We have revised the manuscript following the CONSOR 2010 guidelines as suggested and supplied some information including checklist.

*“Multi-step randomized cluster sampling method was used for subjects’ selection. First, 1-3 districts were selected randomly in the lottery in each city, then the schools were selected randomly in the lottery in each selected district. Eight schools selected randomly were divided into three groups (3 schools for NE, 3 schools for PA and 2 schools as a shared control) in Beijing, and six schools from each other city were randomly sorted into 2 groups (3 for CNP and 3 for control).” （Page 7, Line 130-135）*

*“The present analysis on dietary diversity was not the primary outcome for this trial.* *The calculation of sample size was performed according to the changes of DDS9 in the CNP. The interclass correlation coefficient for DDS9 was 0.18. with a sample size of 4051, we will have 90% power to detect an effect size as much as 0.6 at double side 0.05 level. Our effect size was 0.61, with sample size of 4069, stronger than the minimum detectable level. Only the children in 2nd grade or higher had their dietary records collected, 4933 (both in 5 centers and in Beijing) children with dietary records were extracted from the total participants. Finally, 4846 subjects’ information with both baseline and ending dietary records was used. The trial profile for data analysis is shown in Fig 1.”（Page 8, Line 146-155）*

*“The intra-cluster correlation coefficient was 0.18, 0.13 and 0.13 for DDS9, DDS28 and FVS in CNP study respectively.” （Page 12, Line 231-233）*

*“**The statistical significance level was set at P < 0.05 for overall and P < 0.05/n for subgroup (n means the number of subgroups).” （Page 13, Line 242-243）*

2.2 As a cluster randomised trial conducted separately in different cities in China, it is important to provide more information on the schools recruited to this study and how they were allocated to each group. Was this decided pragmatically or allocated using random numbers? What factors were considered in the selection of schools and were they stratified in randomisation?
Response: The schools were selected randomly in the lottery in each city. We have added the description in the methods *“Multi-step randomized cluster sampling method was used for subjects’ selection. First, 1-3 districts were selected randomly in the lottery in each city, then the schools were selected randomly in the lottery in each selected district.”* (*Page 7, Line 130-132*)

We also supplied the school’s information in the Figure 1 for Trial profile for data analysis. Because we didn’t have the exact number of schools for sample selecting, we couldn’t add the flow chart for schools’ selection.

2.3 As shown in Table 1, the CNP group (15 schools in 5 citie es) received all the NE and PA interventions that was implemented separately in 3 schools in Beijing. With a much larger sample size and comprehensive interventions, the results didn’t show significant effects on the outcomes. In comparison, both NE and PA groups in Beijing schools showed some effects at the end of the trial, especially in the PA group compared to the 2 control schools. Were any process evaluations conducted during the trial that could assess the delivery of different intervention components and the level of protocol compliance?
Response: During the project implementation period, the supervisors of project office went to each center to carry out project supervision to ensure the normal operation of the intervention. After the intervention, the process assessment was also carried out. We found that the intervention was carried out according to the proposal. We also have added the evaluation information about physical activity level. We supplied this information in the methods *“In CNP schools, all above interventions including NE and PA were implemented. In order to ensure the normal operation of the intervention, the supervisors from project office went to each center to carry out project supervision during the program period. The process evaluation indicated that the school carried out as proposal. The physical activity level was measured with energy monitor among 868 students ( 304 in CNP group and 301 in control group in 5 cities, 111 in PA group and 112 in NE group in Beijing).The energy expenditure increased higher in CNP group (77.0 kcal/day) than in control group (63.5 kcal/day, p = 0.967) in five cities study, higher in PA group (96.4 kcal/day) than in NE group (76.7 kcal/day, p = 0.408) in Beijing study.* ” (*Page 9, Line 168-177*)

2.4 Although the present analysis on dietary diversity was not the primary outcome of the trial, it would still be useful to prioritize the outcomes considered in this study and define the minimal effect sizes so that both clinical and statistical significances could be established and used in interpretation.
Response: we have calculated the sample with primary outcome “DDS9”, with a sample size of 4051, we will have 90% power to detective an effect size of as much as 0.6, at double side 0.05 level. With sample size of 4069, our effect size was 0.61, stronger than the minimum detectable level. *“The present analysis on dietary diversity was not the primary outcome for this trial. The calculation of sample size was performed according to the changes of DDS9 in the CNP. The interclass correlation coefficient for DDS9 was 0.18. with a sample size of 4051, we will have 90% power to detect an effect size as much as 0.6 at double side 0.05 level. Our effect size was 0.61, with sample size of 4069, stronger than the minimum detectable level. Only the children in 2nd grade or higher had their dietary records collected, 4933 (both in 5 centers and in Beijing) children with dietary records were extracted from the total participants. Finally, 4846 subjects’ information with both baseline and ending dietary records was used. The trial profile for data analysis is shown in Fig 1.”（Page 8, Line 146-155）*

2.5 To clarify, were the outcomes defined at the end of study or as the change from baseline? Were the subgroup analyses by gender and weight status pre-defined or determined post-hoc? What was the rationale to evaluate the outcomes for breakfast separately? They should be described in the statistical analysis section.
Response: The study outcome was the change of DDS9, DDS28 and FVS from baseline to the end of intervention. Though the gender and weight status were not considered for the sample designing, we think they are very important personal characters influencing the results, so we analyzed the subgroup by gender and weight status. We also pointed out that the results should be treated cautiously for the subgroup analysis in the discussion part. “*At last, the subgroup analysis results should be treated cautiously, because the subgroup was not considered in the sample design.*” (*Page 29, Line 432-433*)

literature studies revealed that the breakfast was more important than other meals (*Ref1, Ref2*), so three meals including breakfast, lunch and supper were evaluated separately as second outcome (described in the method).

*“Literature reveals that the quality of breakfast is more important [Ref1, Ref2] than the others, so the DDS and FVS per meal (breakfast, lunch and supper) were calculated separately as the second outcome indicators.*” (*Page 9, Line 200-202*)

*Ref1: Ptomey LT, Steger FL, Schubert MM, Lee J, Willis EA, Sullivan DK, Szabo-Reed AN, Washburn RA, Donnelly JE. Breakfast Intake and Composition Is Associated with Superior Academic Achievement in Elementary Schoolchildren. J Am Coll Nutr. 2016;35(4):326-33. doi:10.1080/07315724.2015.1048381*

*Ref2: O'Neil CE, Byrd-Bredbenner C, Hayes D, Jana L, Klinger SE, Stephenson-Martin S. The role of breakfast in health: definition and criteria for a quality breakfast. J Acad Nutr Diet. 2014;114(12 Suppl):S8–S26. doi:10.1016/j.jand.2014.08.022*

2.6 For randomised controlled trials, it is not recommended to include baseline outcome measured prior to randomisation as part of the outcome variable assessed post randomisation. As suggested in the EMA guideline on adjustment for baseline covariates in clinical trials, the baseline value of a continuous outcome measure should usually be included as a covariate. This applies whether the outcome variable is defined as the ‘raw outcome’ or as the ‘change from baseline’. In current statistical analysis models, however, a time variable was added with 0 = baseline and 1 = the end of study on the outcome measure with an interaction term between time and treatment group. What terms were used to estimate the intervention effects reported in the table? A more accurate regression model is to fit the outcome measured at the end of the study, with the baseline value and other pre-defined confounders added to the model as covariates for adjustment. The intervention effect is then estimated using the model coefficient for treatment group.
Response: We appreciate for the valuable suggestions, the “change from baseline” was taken as the outcome. For the statistical analysis, we changed the model with general regression models to predict the intervention effect on DDS9, DDS28 and FVS for treatment group as suggestion, and update the results in Table 4 and Table 5. we also revised the description of the model in statistical analysis.

“*The treatment effects were estimated using equation (1) for DDS and FVS and equation (2) for proportions of children consuming certain foods.*

*Y_i_ =* *β_00_ + β_01_Treat_j_ +* *βX_1_ + β_Ƥ_X_Ƥj_ +ε_ij_	(1)*

*Logit (P_ij_) = β_00_ + β_01_Treat_j_ + β_10_Time_ij_ + β_11_Treat_j_×Time_ij_ + β_Ƥ_X_Ƥj_ +ε_ij_	(2)*

*In equation (1), Y_i_ is the outcome of change from baseline to end for child i;* *β_00_ is the intercept parameter; Treat_j_ is an indicator that equals 1 if the student is in the treatment arm and 0 otherwise; X_1_ is the outcome at baseline; and X_Ƥj_ is a vector of control variables at baseline. In equation (2), P_ij_ is the probability of an event for child i at j level; β_00_ is the intercept parameter; Treat_j_ is an indicators that equals 1 if the student is in the treatment arm and 0 otherwise; Time_ij_ is an indicators that equals 1 if the student is at the end of the study and 0 if at the baseline; and X_Ƥj_ is a vector of control variables at baseline. β_11_ reveals the intervention effect in equation (2).*” (*Page13, Line 245-256*)

2.7 As school was the randomisation unit rather than school class, what was the rationale to fit class as the random effect only?

Response: We changed the model used to predict the intervention effect with general regression models as suggestion. And we take school as the randomization unit in the model for the categorized variable(proportion) to run the model and update some results. We revised the description in the text as:

“*The proportions were compared with the generalized linear mixed model (GLMM). The GLMM procedure was used with school as a random-effect variable. The fixed-effect variables included sex, age, parental education level, family income level and intervention type.”* (*Page 12-13, Line 237-241*)

2.8 Please follow the CONSORT 2010 statement with extension to cluster randomised trials in reporting figures and tables. The flow diagram should indicate the follow up schedule, and report both numbers of schools and students in each group. Baseline table should not report p-values, and include both school- and individual-level data collected. The result tables are hard to read with too much information in one table. I would suggest reporting the CNP study and NE/PA study in separate tables, with the randomised groups reported in columns and the outcome measures in rows. Intra-cluster correlation coefficient needs to be reported on each outcome, which is strongly recommended in cluster randomised trials. For the trial conducted in Beijing that included three randomised arms (NE, PA, control), the results for each outcome should be reported together as a multi-arm study rather than two separate analyses for NE vs control and PA vs control.
Response: We appreciate for the good suggestions. We added the school’s information into individual subject flow chart (Figure 1) and deleted the p-values from baseline information tables. For the main result tables including subjects’ characteristics and the results, we have changed into two respectively, one is for the CNP study and the other one is for NE/PA study as suggestion. We also supplied the intra-cluster correlation coefficient for DDS9, DDS28 and FVS in the statically analysis part as *“The intra-cluster correlation coefficient was 0.18, 0.13 and 0.13 for DDS9, DDS28 and FVS in CNP study respectively.” （Page 12, Line 231-233）*

We changed the statistic model with one regression model for three arms in one model together and build two dummy variables for the treat group in Beijing study for the effect evaluation. We have added the information in the manuscript. “*The liner regression model was used for the estimation of the intervention effect on DDS, FVS and the proportion of children consuming different food groups.* *A linear regression model adjusting for confounding factors (weight status, sex, age, parental education level and family income level) was used to compare the changes in continuous variables between the intervention groups and control group.*” (*Page12, Line 233-237*)

2.9 With numerous statistical tests conducted overall and by subgroup, the results must be interpreted with caution as the Type 1 error rate would be inflated without adjustment for multiple comparisons.
Response: We have reset the significant level for the subgroup analysis at 0.05/n, n represented the amount of the subgroups. We added the description in statistical analysis as “*The statistical significance level was set at P < 0.05 for overall and P < 0.05/n for subgroup (n represented the number of subgroups).*” (*Page13, Line 242-243*)

2.10 Based on the results from 3 intervention schools and 2 control schools in Beijing, the title and conclusions focusing only on the significant effects of PA on dietary diversity seem too strong. For example on page 17, line 270, it was reported that the effects on DDS9, DDS28 and FVS were -0.12 (95% CI: -0.38, 0.14; p = 0.365), -0.61 (95% CI: -1.04, -0.17; p = 0.007) and -0.71 (95% CI: -1.35, -0.08; p = 0.028) in the NE group and -0.34 (95% CI: -0.61, -0.08; p = 0.011), -0.73 (95% CI: -1.18, -0.28; p = 0.001) and -1.1 (95% CI: -1.74, -0.45; p = 0.001) in the PA group, respectively. On page 26, line 400, the authors concluded that this result indicated that CNP and NE had no significant effect on the dietary diversity but the dietary diversity decreased in children exposed only to the physical activity intervention. Was this based on DDS9 which was one of the study outcomes? As mentioned earlier, it would be useful to prioritize the outcomes and define the minimal effect sizes so that both clinical and statistical significances could be established and used in interpretation.
Response:

According to the suggestions given by the reviewer as well as other reviewers, we have revised the title to reflect the overall study design, instead of only part of the intervention strategies. We have also reorganized the results accordingly. The title was revised as: *“The effect of comprehensive intervention for childhood obesity on dietary diversity among younger children: Evidence from a school-based randomized controlled trial in China”* (*Page1, Line4-6*)

DDS9 was taken as the primary outcome indicator in our analysis. After getting the reviewing comments, we change to focus on evaluating the effect of CNP mainly, the CNP study was the fundamental part. And the single intervention study evidence for NE and PA was not so sufficient. After reanalyzing the results as suggested, we revised the conclusion as

*“Conclusions*

*Though the comprehensive obesity intervention didn’t improve the overall dietary diversity per day, the positive* *intervention effects were observed on breakfast foods and some foods’ consumption.*” (*Page 4, Line 73-76*)

For the effect size, we have calculated the sample with primary outcome DDS9 according to formula “ n=2(Z_1-α/2_+Z_1-β_)^2^σ^2^(1+(n_i_-1)ρ_1_)/(μ_1_-μ_0_)^2^ ”, define the minimal effect size as 0.6, with 90% power to detect a mean between-group difference in DDS9 of 0.3 times of σ ((μ_1_-μ_0_) =0.3σ), 150 subjects in each school, the needed smallest sample is 4051,which is smaller than the sample size we used (4069). we revised the sample estimation method in the manuscript. “*The calculation of sample size was performed according to the changes of DDS9 in the CNP. The interclass correlation coefficient for DDS9 was 0.18. with a sample size of 4051, we will have 90% power to detective an effect size of as much as 0.6 from 30 schools located in 5 centers, at double side 0.05 level.* *Our effect size was 0.61, with sample size of 4069, stronger than the minimum detectable level. Only the children in 2^nd^ grade or higher had their dietary records collected, 4933 (both in 5 centers and in Beijing) children with dietary records were extracted from the total participants. Finally, 4846 subjects’ information with both baseline and ending dietary records was used. The trial profile for data analysis is shown in Fig 1.*” (*Page8, Line 147-155*)

Response to Reviewer #3: Childhood obesity and overweight are public health problems and represent an important issue in china population. In this regard different interventions (dietary and PA interventions) are designed for children in schools. The study aimed to understand the association of the type of intervention and dietary diversity score among young children. This is an important topic that could be used for planning a proper prevention program in this age group.
The article is well written however, it presents some critical points:
Response: We appreciate the positive comment.

3.1 In the introduction: please add some studies that assess the effect of education or PA on diet quality or diversity.

Response: We added some research as suggested, “*Many researchers found that diet quality could increase after nutrition education intervention among children (ref1), and some studies indicate that a lifestyle intervention plus nutrition education could improve children dietary diversity（ref2）” (Page 6, Lines 101-103)*

*“Though research (ref3) showed that schoolchildren with a high level of physical activity presented a better quality of the diet,* *we wanted to know whether childhood obesity intervention will increase dietary diversity.” (Page6, Lines 115-118)*

*Ref1: Kullen CJ, Farrugia JL, Prvan T, O'Connor HT. Relationship between general nutrition knowledge and diet quality in Australian military personnel. Br J Nutr. 2016;115(8):1489-97. doi:10.1017/S0007114516000532*

*Ref2: Ojeda-Rodríguez A, Zazpe I, Morell-Azanza L, Chueca MJ, Azcona-Sanjulian MC, Marti A. Improved Diet Quality and Nutrient Adequacy in Children and Adolescents with Abdominal Obesity after a Lifestyle Intervention. Nutrients. 2018;10(10):1500. Published 2018 Oct 13. doi:10.3390/nu10101500*

*Ref3: Rosa Guillamon A, Garcia Canto E, Rodríguez García PL, Pérez Soto JJ, Tárraga Marcos ML, Tárraga López PJ. Physical activity, physical fitness and quality of diet in schoolchildren from 8 to 12 years. Nutr Hosp. 2017;34(5):1292-8. doi:10.20960/nh.813*

3.2 from the introduction section, it is not inferred that why the authors assume that the type of obesity programs could affect on DDS differently.

Response: After getting the reviewing comments, we change to focus on evaluating the effect of CNP mainly for this analysis, the CNP study was the fundamental part. And the single intervention study evidence for NE and PA was not so sufficient. After reanalyzing the results as suggested, we revised the conclusion as

*“Conclusions*

*Though the comprehensive obesity intervention didn’t improve the overall dietary diversity per day, the positive intervention effects were observed on breakfast foods and some foods’ consumption.*” (*Page 4, Line 73-76*)

3.3 in the method section, please add the method of randomization.

Response: we have supplied the randomization process as

“*Multi-step randomized cluster sampling method was used for subjects’ selection. First, 1-3 districts were selected randomly in the lottery in each city, then the schools were selected randomly in the lottery in each selected district.*” (*Page 7, Line 130-132*)

3.4. from the method, it is not obvious that how these 38 schools were selected.

Response: the schools were randomly selected in the lottery. We totally agree with the information of schools’ selection is necessary, but it’s very regret that we didn’t have the exact record information during the sampling procedure, so we couldn’t provide the exact numbers for the schools’ selection. We have added the description in the methods as “*First, 1-3 districts were selected randomly in the lottery in each city, then the schools were selected randomly in the lottery in each selected district*”. (*Page7, Line 130-132*)

3.5. it is better to add flow chart for schools selections and in this indicate how many school were invited to take part in these programs and how many accepted it.

Response: We have made a flow chart for the individual subject; we added the school information into individual subject flow chart (Figure 1). We agree that it is better to add flow chart for schools’ selections, because we didn’t have the exact record information of the sampling procedure, so we couldn’t provide this flow chart.

**Fig 1. The trial profile for data analysis**

3.6. IN TABLES: Please the statistical analysis used for comparisons or associations as a table footnote.

Response: we have supplied the table footnote as suggestion.

“*Mixed-effects model was used for comparison, ^**^ p < 0.01, ^*^ p < 0.05.*” in Table2 (*Page 14, Line 275*)

“*Mixed-effects model was used for comparison, ** p < 0.01, * p < 0.05.*” in Table 3 (*Page 15, Line 284*)

*“A linear regression model was used for comparison, the effect was adjusted for weight status, sex, age, parental education level and family income level.”* In Table 4 (*Page 18, Line 302-303*)

*“A linear regression model was used for comparison, the effect was adjusted for sex, age, parental education level and family income level*.” In Table 5 (*Page 21, Line 310-311*)

*“A generalized linear mixed model was used for comparison, the effect was adjusted for sex, age, parental education level and family income level.”* In Table 6 (*Page 24, Line 341-342*)

*“A generalized linear mixed model was used for comparison, the effect was adjusted for sex, age, parental education level and family income level.”* In Table 7 (*Page 25, Line 347-348*)

3.7. Table 3, 4: it seems that the data presented in table 3 and 4 could be merged, so the comparisons become more understandable.

Response: We have merged them together as new Table 4 (*Page 16-18, Line 297-303*)

***Table 4. The outcomes for intervention on DDS and FVS in CNP intervention study (mean ± SD).***

|  |  | ***Control group*** | | ***CNP group*** | |  |  |
| --- | --- | --- | --- | --- | --- | --- | --- |
|  |  | ***Baseline*** | ***Change*** | ***Baseline*** | ***Change*** | ***Effect (95% CI)*** | ***P*** |
| ***For one day*** |  |  |  |  |  |  |  |
| *Total* | *DDS9* | *4.3±1.2* | *-0.2±1.4* | *4.4±1.0* | *-0.2±1.3* | *0.01(-0.05,0.08)* | *0.731* |
|  | *DDS28* | *6.7±2.2* | *-0.5±2.6* | *6.7±1.9* | *-0.5±2.3* | *0.04(-0.08,0.16)* | *0.521* |
|  | *FVS* | *8.7±3.7* | *-0.9±4.3* | *8.5±2.9* | *-0.7±3.3* | *0.08(-0.1,0.26)* | *0.377* |
| *Boys* | *DDS9* | *4.2±1.2* | *-0.2±1.4* | *4.3±1.0* | *-0.3±1.3* | *0(-0.09,0.1)* | *0.982* |
|  | *DDS28* | *6.4±2.1* | *-0.6±2.5* | *6.5±1.9* | *-0.6±2.3* | *0.05(-0.12,0.23)* | *0.569* |
|  | *FVS* | *8.2±3.5* | *-1.0±4.1* | *8.2±2.9* | *-0.9±3.3* | *0.08(-0.17,0.34)* | *0.521* |
| *Girls* | *DDS9* | *4.4±1.2* | *-0.2±1.5* | *4.4±1.0* | *-0.1±1.2* | *0.02(-0.07,0.11)* | *0.641* |
|  | *DDS28* | *6.9±2.2* | *-0.5±2.7* | *6.8±1.9* | *-0.4±2.2* | *0.03(-0.14,0.19)* | *0.76* |
|  | *FVS* | *9.1±3.8* | *-0.9±4.5* | *8.8±2.9* | *-0.6±3.3* | *0.07(-0.17,0.32)* | *0.567* |
| *Normal weight & thinness* | *DDS9* | *4.3±1.2* | *-0.2±1.4* | *4.4±1.0* | *-0.2±1.3* | *0.01(-0.07,0.08)* | *0.871* |
|  | *DDS28* | *6.7±2.2* | *-0.6±2.6* | *6.7±1.9* | *-0.5±2.3* | *0.01(-0.12,0.14)* | *0.887* |
|  | *FVS* | *8.8±3.7* | *-1.0±4.3* | *8.6±2.9* | *-0.7±3.3* | *0.02(-0.17,0.22)* | *0.812* |
| *Overweight & obesity* | *DDS9* | *4.1±1.1* | *-0.2±1.4* | *4.3±1.1* | *-0.2±1.3* | *0.03(-0.11,0.18)* | *0.669* |
|  | *DDS28* | *6.4±2.1* | *-0.4±2.7* | *6.7±1.9* | *-0.4±2.2* | *0.14(-0.12,0.4)* | *0.285* |
|  | *FVS* | *8.1±3.5* | *-0.7±4.3* | *8.4±3.0* | *-0.6±3.3* | *0.28(-0.11,0.66)* | *0.165* |
| ***For single meal*** |  |  |  |  |  |  |  |
| *Breakfast* | *DDS9* | *1.8±0.7* | *-0.1±0.9* | *1.9±0.7* | *0±0.8* | *0.09(0.05,0.13)* | *<.0001* |
|  | *DDS28* | *2.2±0.8* | *-0.1±1.1* | *2.2±0.8* | *-0.1±0.9* | *0.05(0,0.11)* | *0.049* |
|  | *FVS* | *2.3±0.9* | *-0.2±1.2* | *2.3±0.8* | *-0.1±1.0* | *0.07(0.01,0.12)* | *0.024* |
| *Lunch* | *DDS9* | *2.1±0.7* | *-0.1±0.9* | *2.1±0.7* | *0±0.9* | *-0.07(-0.12,-0.02)* | *0.01* |
|  | *DDS28* | *2.4±1* | *-0.2±1.2* | *2.4±0.9* | *-0.1±1.1* | *-0.1(-0.18,-0.03)* | *0.007* |
|  | *FVS* | *2.6±1.1* | *-0.3±1.4* | *2.5±1* | *-0.1±1.2* | *-0.14(-0.23,-0.05)* | *0.002* |
| *Supper* | *DDS9* | *2.8±0.9* | *-0.1±1.1* | *2.8±0.7* | *-0.1±0.9* | *0.01(-0.04,0.17)* | *0.625* |
|  | *DDS28* | *3.5±1.3* | *-0.2±1.7* | *3.5±1.1* | *-0.3±1.4* | *0.01(-0.07,0.09)* | *0.81* |
|  | *FVS* | *3.9±1.8* | *-0.3±2.2* | *3.8±1.4* | *-0.3±1.6* | *0.03(-0.06,0.13)* | *0.486* |

*CNP, comprehensive intervention including nutrition education and physical activity; DDS, Dietary Diversity Score; DDS9: Dietary Diversity Score for 9 food groups; DDS28: Dietary Diversity Score for 28 food groups; FVS, Food Variety Score.*

*A linear regression model was used for comparison,* *the effect was adjusted for weight status, sex, age, parental education level and family income level.*

3.8. Table 5, 6: it seems that the data presented in table 5 and 6 could be merged, so the comparisons become more understandable

Response: We have merged them together as new Table 5 (*Page18-21, Line 305-311*)

***Table 5. The outcomes of the intervention on DDS and FVS in NE or PA intervention study (mean ± SD)****.*

|  |  | ***Control group*** | | ***NE group*** | | | | ***PA group*** | | | |
| --- | --- | --- | --- | --- | --- | --- | --- | --- | --- | --- | --- |
|  |  | ***Baseline*** | ***Change*** | ***Baseline*** | ***Change*** | ***Effect (95% CI)*** | ***P*** | ***Baseline*** | ***Change*** | ***Effect (95% CI)*** | ***P*** |
| ***For one day*** |  |  |  |  |  |  |  |  |  |  |  |
| *Total* | *DDS9* | *4.0±1.2* | *0.1±1.5* | *4.3±1.1* | *0.1±1.1* | *0.06(-0.14,0.26)* | *0.567* | *4.1±1.0* | *-0.2±1.2* | *-0.27(-0.47,-0.06)* | *0.01* |
|  | *DDS28* | *5.8±2.1* | *0.6±2.8* | *6.9±1.9* | *0.1±1.9* | *0.02(-0.33,0.38)* | *0.893* | *6.2±1.7* | *0±1.9* | *-0.55(-0.91,-0.19)* | *0.003* |
|  | *FVS* | *6.9±3.1* | *0.7±4.1* | *8.6±3.1* | *0.2±2.8* | *0.23(-0.29,0.76)* | *0.386* | *7.6±2.3* | *-0.2±2.6* | *-0.74(-1.27,-0.22)* | *0.006* |
| *Boys* | *DDS9* | *3.9±1.2* | *0.1±1.5* | *4.3±1.0* | *-0.1±1.2* | *0.09(-0.2,0.39)* | *0.542* | *4.0±1.0* | *-0.2±1.2* | *-0.28(-0.58,0.02)* | *0.068* |
|  | *DDS28* | *5.7±2.2* | *0.4±2.8* | *6.9±1.9* | *-0.2±2.0* | *0.07(-0.44,0.57)* | *0.793* | *5.9±1.7* | *-0.2±1.8* | *-0.49(-0.99,0.01)* | *0.054* |
|  | *FVS* | *6.8±3.1* | *0.5±3.8* | *8.6±3.2* | *-0.3±3.1* | *0.4(-0.33,1.12)* | *0.284* | *7.2±2.3* | *-0.5±2.5* | *-0.64(-1.36,0.07)* | *0.079* |
| *Girls* | *DDS9* | *4.2±1.1* | *0.2±1.4* | *4.3±1.1* | *0.1±1.1* | *0.06(-0.22,0.33)* | *0.695* | *4.3±1.0* | *-0.1±1.2* | *-0.22(-0.51,0.06)* | *0.12* |
|  | *DDS28* | *6.0±2.0* | *0.8±2.8* | *6.9±1.9* | *0.3±1.8* | *0.02(-0.5,0.53)* | *0.951* | *6.4±1.7* | *0.1±1.9* | *-0.57(-1.09,-0.05)* | *0.033* |
|  | *FVS* | *7.1±3.1* | *1.0±4.4* | *8.6±3.0* | *0.5±2.5* | *0.14(-0.63,0.92)* | *0.72* | *7.9±2.3* | *0±2.7* | *-0.8(-1.58,-0.02)* | *0.046* |
| *Normal weight & thinness* | *DDS9* | *4±1.2* | *0.2±1.5* | *4.3±1.0* | *0.1±1.1* | *0.06(-0.15,0.28)* | *0.578* | *4.1±1.0* | *-0.1±1.2* | *-0.33(-0.55,-0.11)* | *0.003* |
|  | *DDS28* | *5.9±2.2* | *0.7±2.9* | *6.9±1.9* | *0.1±1.9* | *-0.01(-0.4,0.37)* | *0.955* | *6.1±1.7* | *-0.1±1.8* | *-0.71(-1.09,-0.32)* | *0* |
|  | *FVS* | *7.0±3.3* | *0.8±4.3* | *8.6±3.1* | *0.2±2.8* | *0.19(-0.38,0.76)* | *0.515* | *7.5±2.4* | *-0.3±2.5* | *-0.94(-1.52,-0.37)* | *0.001* |
| *Overweight & obesity* | *DDS9* | *4.2±0.9* | *-0.3±1.3* | *4.4±1.1* | *-0.2±1.3* | *0.29(-0.27,0.86)* | *0.309* | *4.4±1.1* | *-0.2±1.4* | *0.21(-0.35,0.78)* | *0.457* |
|  | *DDS28* | *5.6±1.2* | *0±1.9* | *6.8±2.0* | *-0.2±2.0* | *0.77(-0.22,1.76)* | *0.126* | *6.3±1.7* | *0.1±2.3* | *0.66(-0.31,1.63)* | *0.183* |
|  | *FVS* | *6.5±1.6* | *-0.2±2.5* | *8.7±2.9* | *-0.2±2.9* | *1.28(-0.21,2.76)* | *0.091* | *7.8±2.3* | *-0.2±3.0* | *0.83(-0.61,2.28)* | *0.257* |
| ***For single meal*** |  |  |  |  |  |  |  |  |  |  |  |
| *Breakfast* | *DDS9* | *1.7±0.7* | *0.2±1.0* | *2.1±0.7* | *0.1±0.9* | *0.06(-0.12,0.24)* | *0.508* | *1.9±0.7* | *0.1±1.0* | *-0.04(-0.22,0.14)* | *0.658* |
|  | *DDS28* | *2.1±0.9* | *0.3±1.2* | *2.7±0.8* | *0.1±1.0* | *0.12(-0.09,0.33)* | *0.275* | *2.2±0.7* | *0.1±1.1* | *-0.03(-0.25,0.18)* | *0.769* |
|  | *FVS* | *2.1±0.9* | *0.3±1.3* | *2.8±1.0* | *0.1±1.0* | *0.07(-0.16,0.31)* | *0.554* | *2.3±0.8* | *0.1±1.1* | *-0.08(-0.32,0.15)* | *0.489* |
| *Lunch* | *DDS9* | *2.1±0.8* | *0.2±1.1* | *2.4±0.7* | *0±0.9* | *0.27(0.1,0.44)* | *0.002* | *2.2±0.8* | *0.1±1.1* | *-0.06(-0.23,0.11)* | *0.491* |
|  | *DDS28* | *2.3±1* | *0.3±1.5* | *2.9±0.9* | *0±1* | *0.28(0.06,0.51)* | *0.013* | *2.5±0.9* | *0.2±1.3* | *-0.18(-0.41,0.04)* | *0.112* |
|  | *FVS* | *2.4±1.1* | *0.4±1.6* | *3.1±1.1* | *-0.1±1.1* | *0.47(0.18,0.76)* | *0.001* | *2.5±0.9* | *0.2±1.3* | *-0.22(-0.51,0.07)* | *0.14* |
| *Supper* | *DDS9* | *2.5±0.9* | *0.1±1.3* | *2.7±1* | *0.2±1* | *0.15(-0.02,0.32)* | *0.076* | *2.5±0.8* | *0±1* | *-0.21(-0.39,-0.04)* | *0.013* |
|  | *DDS28* | *3±1.2* | *0.2±1.6* | *3.3±1.2* | *0.2±1.3* | *0.24(0.01,0.47)* | *0.044* | *3.1±1* | *-0.1±1.2* | *-0.33(-0.56,-0.09)* | *0.007* |
|  | *FVS* | *3.2±1.5* | *0.2±2.1* | *3.7±1.6* | *0.3±1.7* | *0.22(-0.06,0.49)* | *0.12* | *3.4±1.2* | *-0.1±1.5* | *-0.40(-0.68,-0.12)* | *0.004* |

*DDS, Dietary Diversity Score; DDS9: Dietary Diversity Score for 9 food groups; DDS28: Dietary Diversity Score for 28 food groups; FVS, Food Variety Score; NE, nutrition education intervention; PA, physical activity intervention.*

*A linear regression model was used for comparison, the effect was adjusted for weight status, sex, age, parental education level and family income level.*

3.9. Why the authors analysed the data secretively for breakfast?

Response: literature studies revealed that the breakfast was more important than other meals (ref1, ref2), so three meals including breakfast, lunch and supper were evaluated separately as second outcome (described in the method).

*“Literature reveals that the quality of breakfast is more important (ref1, ref2) than the others, so the DDS and FVS per meal (breakfast, lunch and supper) were calculated separately as the second outcome indicators.*” (*Page 11, Line 200-202*)

*Ref1: Ptomey LT, Steger FL, Schubert MM, et al. Breakfast Intake and Composition Is Associated with Superior Academic Achievement in Elementary Schoolchildren. J Am Coll Nutr. 2016;35(4):326-33. doi:10.1080/07315724.2015.1048381*

*Ref2: O'Neil CE, Byrd-Bredbenner C, Hayes D, Jana L, Klinger SE, Stephenson-Martin S. The role of breakfast in health: definition and criteria for a quality breakfast. J Acad Nutr Diet. 2014;114(12 Suppl):S8–S26. doi:10.1016/j.jand.2014.08.022*

3.10. the second paragraph of discussion is not related to this part. it may be more suitable for introduction section.

Response: we have adjusted this paragraph as suggested. One part was removed into the introduction *“And we observed a moderately significant effect on the combined prevalence of overweight and obesity, which increased by 1.5 percent in the control group and 0.2 percent in the CNP group after intervention. The effect was significantly stronger among girls than boys (-1.4% vs. -0.9%). However, we did not find a significant effect in the nutrition education group or the physical activity group. Several school-based interventions for childhood obesity have revealed a positive effect on changing eating behaviors and improving vegetable and fruit consumption and the availability of healthy foods, but few have focused on dietary diversity or food variety.”* (*Page 6, Line 107-115*)

the other part was removed into the end of the first paragraph of discussion *“By analyzing different food consumption, the proportions of children increased more for fruits and decreased less for cereals in the CNP group than the control group after the intervention. The cereals and fruits were the important energy and micronutrient sources, so these findings revealed that these consumption behavior changes may lead to the improvement of diet quality in comprehensive intervention groups.”* (*Page26, Line 356-361*)

3.11. in the discussion section it is recommended to discuss the finding of the present study. In this part, the author mostly discussed about obesity, not diet quality and diversity.

Response: we have added the discussions about diet quality and nutrition education, such as *“The breakfast is generally taken as the most important meal of the day and is purported to confer a number of benefits for diet quality, health and academic performance. Study reveals that children who habitually consume high quality breakfast are more likely to have better nutrient intake [30]. So, the improvement of dietary diversity and food variety at breakfast could increase the diet quality and benefit the children.”* (*Page26, Line 361-366*)

*“It has been demonstrated that both nutrition education and lifestyle intervention could improve the dietary diversity among children or adults [15,34].”* (*Page27, Line 379-380*)
