# Supplementary material for: The effect of comprehensive intervention for childhood obesity on dietary diversity among younger children: Evidence from a school-based randomized controlled trial in China
Source: PLoS One. 2020 Jul 17;15(7):e0235951. doi: 10.1371/journal.pone.0235951 (PMC7367455; doi:10.1371/journal.pone.0235951)
Supplement: Attachment — Submitted filename: Response to Reviewers.docx [file pone.0235951.s006.docx]

7 May 2020

Dear Dr. Seth Adu-Afarwuah, Editors and Reviewers:

We very much appreciate your thoughtful comments on our manuscript (PONE-D-19-24892R1, “The effect of comprehensive intervention for childhood obesity on dietary diversity among younger children: Evidence from a school-based randomized controlled trial in China”). We have carefully considered each comment and responded point-by-point below. We have made corresponding changes in the manuscript with tracked changes highlighting both additions and deletions.

In addition, we have carefully checked the format requirement and revised the manuscript according to the requirement of *PLOS ONE*.

We believe that your comments and our responses to them have improved our manuscript considerably, and we hope you find our revised manuscript suitable for publication.

Sincerely,

Guansheng Ma Prof.

Department of Nutrition and Food Hygiene,

School of Public Health, Peking University,

38 Xue Yuan Road,

Beijing, China.

Email: [mags@bjmu.edu.cn](mailto:mags@bjmu.edu.cn)

<Tel:13801080336>,

Fax: +86 10 82105184.

Guansheng Ma Prof.

Department of Nutrition and Food Hygiene,

School of Public Health, Peking University,

38 Xue Yuan Road,

Beijing, China.

Email: [mags@bjmu.edu.cn](mailto:mags@bjmu.edu.cn)

<Tel:13801080336>,

Fax: +86 10 82105184.

Response to Journal Requirements:

In your revised version, please format the tables properly, so that they can be read more easily by reviewers.

Response: We have revised the format of tables according to PLOS ONE’s style.

Reviewer #1: (No Response)

Responses to Reviewer #2:

**2.1** Thank you for the revised paper and responses to all the comments. With further clarification on the original trial design, my concerns remain on the overall conduct of the two studies and the analysis results reported using the specified models.
**Response:** Thanks a lot for the comments.

**2.2** As shown in Table 1, the two separate cluster randomised trials conducted in Beijing and other 5 cities used same intervention components on PA and NE (separately or combined). The authors stated that all the schools implemented the programme as proposed. If this was the case, it would be hard to evaluate the intervention effects with observed differences on the demographic characteristics and estimated effect sizes between two studies. There are also large geographic differences between these cities in China, and how similar the selected schools were between groups is unknown. If the CNP study was the fundamental part of the trial, the authors should perhaps focus on this main study only and report the change on both BMI (primary outcome) and dietary diversity in the paper.
**Response:** We appreciate the valuable suggestions from the review expert. We quite agree that there are some geographic differences between these cities in China. In order to decrease the difference, these schools were selected according to some inclusion criteria: 1) non-boarding school; 2) the prevalence of obesity, based on the routine physical examination records, was above 10%; 3) Providing school lunch feeding, and more than 50% students have lunch at school. which we have provided in the published paper (*Ref1*), and we also supplied this information in this manuscript. We think these similar characters make the intervention and evaluation more scientific. As the reviewer mentioned that the CNP study was the fundamental part of the trial. We have put our results focused on this main study in the first-round revision. For the single PA and NE intervention, these results were reported to assist in explaining the other part based on the overall perspective of the trial. This study was not the primary analysis of this trial. The effect on the BMI has been analyzed and published before (*Ref1*). In this study, we analyzed the effect focused on dietary diversity, so the BMI was just taken as the baseline characteristic not the outcome.

*Ref1*: Xu H, Li Y, Zhang Q, Hu X, Liu A, Du S, et al. Comprehensive school-based intervention to control overweight and obesity in China: a cluster randomized controlled trial. Asia Pac J Clin Nutr. 2017; 26(6):1139-51.

We supplied the information in the Materials and methods

“*The schools were selected according to some inclusion criteria: 1) non-boarding school; 2) the prevalence of obesity, based on the routine physical examination records, was above 10%; 3) Providing school lunch feeding, and more than 50% students have lunch at school*.” ***Page 7, L212-215.***

**2.3** For a cluster randomised trial, the unit of randomisation is at the cluster level while the data collection may include both cluster and individual participants’ data. The regression models should consider the within- and between-cluster variations, and fit both fixed and random effects using the mixed models. Fitting baseline outcome value as a covariate in the fixed effect model is applicable to both continuous and categorical outcomes, not just the change from baseline scores. The revised equation (1) was fitted using a standard linear regression model without a random effect. The equation (2) fitted the baseline value as part of the outcome measures in the GLMM model. When an interaction term was added in the logistic model, the beta coefficient for the interaction term should not be interpreted independently from the main terms on treatment and time. The model-adjusted group difference needs to be estimated at the end of the study using the odds ratio (i.e. exponential of the raw model estimate).
**Response:** We appreciated the valuable suggestions from the reviewer and revised the equation (1) into the mixed model, the Cluster was taken into the model as the random effect. We also revised the equation (2) as suggested. The main results were updated in Table 4 -Table7 and results in the manuscript, we changed the beta coefficient into odds ratio, which was shown in Table 6 and Table 7.

The description was revised in the manuscript as followed:

**For the models, as**

“*Y_ij_ = γ_00_ +β_0_Cluster+ β_01_Treat + β_1_X_1ij_ + β_Ƥ_X_Ƥij_ +ε_ij_	(1)*

*Logit (P_ij_) = γ_00_ +β_0_Cluster + β_01_Treat + β_1_X_1ij_ + β_Ƥ_X_Ƥij_ +ε_ij_	(2)*

*In equation (1), Y_ij_ is the outcome of change from baseline to end for child i at level j; γ_00_ is the intercept parameter; Cluster is random effect indicator; Treat is an indicator that equals 1 if the student is in the treatment arm and 0 otherwise; X_1ij_ is the outcome at baseline and X_Ƥij_ is a vector of control variables at baseline. In equation (2), P_ij_ is the change of probability of an event from baseline to end for child i at level j; β_00_ is the intercept parameter; Cluster is random effect indicator; Treat is an indicator that equals 1 if the student is in the treatment arm and 0 otherwise; and X_Ƥij_ is a vector of control variables at baseline. β_01_ reveals the intervention effect.*” ***Page 13, L400-409***

**For the updated results, as**

“*The intervention effects per day of comprehensive intervention group were 0 (95% Confidence Interval (CI): 0, 0.1; p = 0.382) on DDS9, 0.1 (95% CI: -0.1, 0.2; p = 0.374) on DDS28 and 0.1 (95% CI: -0.1, 0.3; p = 0.186) on FVS of overall diet, which was 0.1 (95% CI: 0, 0.1; p < 0.001) on DDS9, 0 (95% CI: 0, 0.1; p = 0.168) on DDS28 and 0.1 (95% CI: 0, 0.1; p = 0.067) on FVS of dietary scores of breakfast only. Additionally, CNP group had greater increases in cereals, meat and fruits, and more decreases in eggs, fish and dried legumes consumption proportions as compared with the control group.*” ***in abstract Page 3, L68-84.***

“*The intervention effects on DDS and FVS according to dietary intake are shown in Table 4 for CNP study and in Table 5 for NE or PA study. Compared with the control group, the effects of CNP were 0 (95% Confidence Interval (CI): 0, 0.1; p = 0.382) on DDS9, 0.1 (95% CI: -0.1, 0.2; p = 0.374) on DDS28 and 0.1 (95% CI: -0.1, 0.3; p = 0.186) on FVS. No significant difference of intervention effects was found across subgroups. When we evaluated the dietary scores based on the breakfast foods only, positive intervention effects of CNP group were observed significant on DDS9 (0.1; 95% CI: 0, 0.1; p < 0.001), and nonsignificant on DDS28 (0; 95% CI: 0, 0.1; p = 0.168) and FVS (0.1; 95% CI: 0, 0.1; p = 0.067).* ” ***Page 16, L486-494***

“*The effects on DDS9, DDS28 and FVS were 0.1 (95% CI: -0.1,0.3; p =0.294), 0.1 (95% CI: -0.3, 0.4; p =0.747) and 0.4 (95% CI: -0.2,0.9; p =0.201) in the NE group and -0.1 (95% CI: -0.2, 0; p =0.037), -0.3 (95% CI: -0.4,-0.1; p =0.005) and -0.3 (95% CI: -0.6, 0; p =0.021) in the PA group, respectively. No significant effects on DDS or FVS were found at breakfast in NE group or PA group.*” ***Page 22,*** ***L535-539***

**For the updated tables of outcomes, as**

**“*****Table 4. The outcomes for intervention on DDS and FVS in CNP intervention study (mean ± SD).***

| ***Group*** | ***Variable*** | ***Control group*** | | ***CNP group*** | |  |  |
| --- | --- | --- | --- | --- | --- | --- | --- |
|  |  | ***Baseline*** | ***Change*** | ***Baseline*** | ***Change*** | ***Effect (95% CI)*** | ***P-value*** |
| ***For one day*** |  |  |  |  |  |  |  |
| *Total* | *DDS9* | *4.3±1.2* | *-0.2±1.4* | *4.4±1.0* | *-0.2±1.3* | *0 (0, 0.1)* | *0.382* |
|  | *DDS28* | *6.7±2.2* | *-0.5±2.6* | *6.7±1.9* | *-0.5±2.3* | *0.1(-0.1, 0.2)* | *0.374* |
|  | *FVS* | *8.7±3.7* | *-0.9±4.3* | *8.5±2.9* | *-0.7±3.3* | *0.1 (-0.1, 0.3)* | *0.186* |
| *Boys* | *DDS9* | *4.2±1.2* | *-0.2±1.4* | *4.3±1.0* | *-0.3±1.3* | *0 (-0.1, 0.1)* | *0.807* |
|  | *DDS28* | *6.4±2.1* | *-0.6±2.5* | *6.5±1.9* | *-0.6±2.3* | *0.1 (-0.1, 0.2)* | *0.453* |
|  | *FVS* | *8.2±3.5* | *-1.0±4.1* | *8.2±2.9* | *-0.9±3.3* | *0.1 (-0.1, 0.4)* | *0.324* |
| *Girls* | *DDS9* | *4.4±1.2* | *-0.2±1.5* | *4.4±1.0* | *-0.1±1.2* | *0 (0, 0.1)* | *0.334* |
|  | *DDS28* | *6.9±2.2* | *-0.5±2.7* | *6.8±1.9* | *-0.4±2.2* | *0 (-0.1, 0.2)* | *0.643* |
|  | *FVS* | *9.1±3.8* | *-0.9±4.5* | *8.8±2.9* | *-0.6±3.3* | *0.1 (-0.1, 0.3)* | *0.411* |
| *Normal weight & thinness* | *DDS9* | *4.3±1.2* | *-0.2±1.4* | *4.4±1.0* | *-0.2±1.3* | *0.1 (-0.1, 0.2)* | *0.488* |
|  | *DDS28* | *6.7±2.2* | *-0.6±2.6* | *6.7±1.9* | *-0.5±2.3* | *0.2 (-0.1, 0.4)* | *0.162* |
|  | *FVS* | *8.8±3.7* | *-1.0±4.3* | *8.6±2.9* | *-0.7±3.3* | *0.3 (0, 0.7)* | *0.086* |
| *Overweight & obesity* | *DDS9* | *4.1±1.1* | *-0.2±1.4* | *4.3±1.1* | *-0.2±1.3* | *0 (0, 0.1)* | *0.525* |
|  | *DDS28* | *6.4±2.1* | *-0.4±2.7* | *6.7±1.9* | *-0.4±2.2* | *0 (-0.1, 0.1)* | *0.836* |
|  | *FVS* | *8.1±3.5* | *-0.7±4.3* | *8.4±3.0* | *-0.6±3.3* | *0.1(-0.1, 0.2)* | *0.608* |
| ***For single meal*** |  |  |  |  |  |  |  |
| *Breakfast* | *DDS9* | *1.8±0.7* | *-0.1±0.9* | *1.9±0.7* | *0±0.8* | *0.1 (0, 0.1)* | *<0.001* |
|  | *DDS28* | *2.2±0.8* | *-0.1±1.1* | *2.2±0.8* | *-0.1±0.9* | *0 (0, 0.1)* | *0.168* |
|  | *FVS* | *2.3±0.9* | *-0.2±1.2* | *2.3±0.8* | *-0.1±1.0* | *0.1 (0, 0.1)* | *0.067* |
| *Lunch* | *DDS9* | *2.1±0.7* | *-0.1±0.9* | *2.1±0.7* | *0±0.9* | *-0.1 (-0.1,0)* | *0.045* |
|  | *DDS28* | *2.4±1* | *-0.2±1.2* | *2.4±0.9* | *-0.1±1.1* | *-0.1 (-0.2, 0)* | *0.022* |
|  | *FVS* | *2.6±1.1* | *-0.3±1.4* | *2.5±1* | *-0.1±1.2* | *-0.1 (-0.2, 0)* | *0.007* |
| *Supper* | *DDS9* | *2.8±0.9* | *-0.1±1.1* | *2.8±0.7* | *-0.1±0.9* | *0 (0, 0.1)* | *0.162* |
|  | *DDS28* | *3.5±1.3* | *-0.2±1.7* | *3.5±1.1* | *-0.3±1.4* | *0 (0, 0.1)* | *0.378* |
|  | *FVS* | *3.9±1.8* | *-0.3±2.2* | *3.8±1.4* | *-0.3±1.6* | *0.1 (0, 0.2)* | *0.118* |

*CI, Confidence Interval; CNP, comprehensive intervention including nutrition education and physical activity; DDS, Dietary Diversity Score; DDS9: Dietary Diversity Score for 9 food groups; DDS28: Dietary Diversity Score for 28 food groups; FVS, Food Variety Score.*

*A linear mixed model was used for comparison,* *the effect was adjusted for weight status, sex, age, parental education level and family income level.*

***Table 5. The outcomes of the intervention on DDS and FVS in NE or PA intervention study (mean ± SD)****.*

| ***Group*** | ***Variable*** | ***Control group*** | | ***NE group*** | | | | ***PA group*** | | | |
| --- | --- | --- | --- | --- | --- | --- | --- | --- | --- | --- | --- |
|  |  | ***Baseline*** | ***Change*** | ***Baseline*** | ***Change*** | ***Effect (95% CI)*** | ***P-value*** | ***Baseline*** | ***Change*** | ***Effect (95% CI)*** | ***P-value*** |
| ***For one day*** |  |  |  |  |  |  |  |  |  |  |  |
| *Total* | *DDS9* | *4.0±1.2* | *0.1±1.5* | *4.3±1.1* | *0.1±1.1* | *0.1 (-0.1, 0.3)* | *0.294* | *4.1±1.0* | *-0.2±1.2* | *-0.1 (-0.2, 0)* | *0.037* |
|  | *DDS28* | *5.8±2.1* | *0.6±2.8* | *6.9±1.9* | *0.1±1.9* | *0.1 (-0.3, 0.4)* | *0.747* | *6.2±1.7* | *0±1.9* | *-0.3 (-0.4, -0.1)* | *0.005* |
|  | *FVS* | *6.9±3.1* | *0.7±4.1* | *8.6±3.1* | *0.2±2.8* | *0.4 (-0.2, 0.9)* | *0.201* | *7.6±2.3* | *-0.2±2.6* | *-0.3 (-0.6, 0)* | *0.021* |
| *Boys* | *DDS9* | *3.9±1.2* | *0.1±1.5* | *4.3±1.0* | *-0.1±1.2* | *0.1 (-0.2, 0.4)* | *0.381* | *4.0±1.0* | *-0.2±1.2* | *-0.1 (-0.3, 0)* | *0.147* |
|  | *DDS28* | *5.7±2.2* | *0.4±2.8* | *6.9±1.9* | *-0.2±2.0* | *0.1(-0.4, 0.6)* | *0.64* | *5.9±1.7* | *-0.2±1.8* | *-0.2 (-0.5, 0)* | *0.103* |
|  | *FVS* | *6.8±3.1* | *0.5±3.8* | *8.6±3.2* | *-0.3±3.1* | *0.5 (-0.2, 1.3)* | *0.162* | *7.2±2.3* | *-0.5±2.5* | *-0.2 (-0.6, 0.1)* | *0.187* |
| *Girls* | *DDS9* | *4.2±1.1* | *0.2±1.4* | *4.3±1.1* | *0.1±1.1* | *0.1 (-0.2, 0.4)* | *0.431* | *4.3±1.0* | *-0.1±1.2* | *-0.1 (-0.2, 0.1)* | *0.218* |
|  | *DDS28* | *6.0±2.0* | *0.8±2.8* | *6.9±1.9* | *0.3±1.8* | *0 (-0.5, 0.6)* | *0.892* | *6.4±1.7* | *0.1±1.9* | *-0.3 (-0.5, 0)* | *0.042* |
|  | *FVS* | *7.1±3.1* | *1.0±4.4* | *8.6±3.0* | *0.5±2.5* | *0.3 (-0.6, 1.1)* | *0.527* | *7.9±2.3* | *0±2.7* | *-0.4 (-0.8, 0)* | *0.081* |
| *Normal weight & thinness* | *DDS9* | *4±1.2* | *0.2±1.5* | *4.3±1.0* | *0.1±1.1* | *0.4 (-0.2, 1.0)* | *0.162* | *4.1±1.0* | *-0.1±1.2* | *0.1 (-0.1, 0.4)* | *0.286* |
|  | *DDS28* | *5.9±2.2* | *0.7±2.9* | *6.9±1.9* | *0.1±1.9* | *1.0 (0, 2.0)* | *0.057* | *6.1±1.7* | *-0.1±1.8* | *0.4 (-0.1, 0.9)* | *0.093* |
|  | *FVS* | *7.0±3.3* | *0.8±4.3* | *8.6±3.1* | *0.2±2.8* | *1.6 (0.1, 3.2)* | *0.041* | *7.5±2.4* | *-0.3±2.5* | *0.5 (-0.2, 1.2)* | *0.135* |
| *Overweight & obesity* | *DDS9* | *4.2±0.9* | *-0.3±1.3* | *4.4±1.1* | *-0.2±1.3* | *0.1 (-0.1, 0.3)* | *0.519* | *4.4±1.1* | *-0.2±1.4* | *-0.2 (-0.3,0)* | *0.006* |
|  | *DDS28* | *5.6±1.2* | *0±1.9* | *6.8±2.0* | *-0.2±2.0* | *-0.1 (-0.5, 0.3)* | *0.719* | *6.3±1.7* | *0.1±2.3* | *-0.4 (-0.6, -0.2)* | *0* |
|  | *FVS* | *6.5±1.6* | *-0.2±2.5* | *8.7±2.9* | *-0.2±2.9* | *0.2 (-0.4, 0.8)* | *0.569* | *7.8±2.3* | *-0.2±3.0* | *-0.5 (-0.8, -0.2)* | *< 0.001* |
| ***For single meal*** |  |  |  |  |  |  |  |  |  |  |  |
| *Breakfast* | *DDS9* | *1.7±0.7* | *0.2±1.0* | *2.1±0.7* | *0.1±0.9* | *0.1 (-0.1, 0.3)* | *0.44* | *1.9±0.7* | *0.1±1.0* | *0 (-0.1, 0.1)* | *0.754* |
|  | *DDS28* | *2.1±0.9* | *0.3±1.2* | *2.7±0.8* | *0.1±1.0* | *0.2 (0, 0.4)* | *0.126* | *2.2±0.7* | *0.1±1.1* | *0 (-0.1, 0.1)* | *0.903* |
|  | *FVS* | *2.1±0.9* | *0.3±1.3* | *2.8±1.0* | *0.1±1.0* | *0.2 (-0.1, 0.4)* | *0.155* | *2.3±0.8* | *0.1±1.1* | *0 (-0.1, 0.1)* | *0.973* |
| *Lunch* | *DDS9* | *2.1±0.8* | *0.2±1.1* | *2.4±0.7* | *0±0.9* | *0.3 (0.2, 0.5)* | *0* | *2.2±0.8* | *0.1±1.1* | *0 (-0.1, 0.1)* | *0.94* |
|  | *DDS28* | *2.3±1* | *0.3±1.5* | *2.9±0.9* | *0±1* | *0.3 (0, 0.5)* | *0.03* | *2.5±0.9* | *0.2±1.3* | *-0.1 (-0.2, 0)* | *0.082* |
|  | *FVS* | *2.4±1.1* | *0.4±1.6* | *3.1±1.1* | *-0.1±1.1* | *0.5 (0.2, 0.8)* | *0.001* | *2.5±0.9* | *0.2±1.3* | *-0.1 (-0.2, 0.1)* | *0.197* |
| *Supper* | *DDS9* | *2.5±0.9* | *0.1±1.3* | *2.7±1* | *0.2±1* | *0.2 (0, 0.4)* | *0.029* | *2.5±0.8* | *0±1* | *-0.1 (-0.2, 0)* | *0.046* |
|  | *DDS28* | *3±1.2* | *0.2±1.6* | *3.3±1.2* | *0.2±1.3* | *0.3 (0, 0.5)* | *0.018* | *3.1±1* | *-0.1±1.2* | *-0.1 (-0.3, 0)* | *0.023* |
|  | *FVS* | *3.2±1.5* | *0.2±2.1* | *3.7±1.6* | *0.3±1.7* | *0.3 (0,0.6)* | *0.054* | *3.4±1.2* | *-0.1±1.5* | *-0.2 (-0.3, 0)* | *0.017* |

*CI, Confidence Interval; DDS, Dietary Diversity Score; DDS9: Dietary Diversity Score for 9 food groups; DDS28: Dietary Diversity Score for 28 food groups; FVS, Food Variety Score; NE, nutrition education intervention; PA, physical activity intervention.*

*A linear mixed model was used for comparison, the effect was adjusted for weight status, sex, age, parental education level and family income level.*

***Table 6. The proportion of children consuming different food groups in CNP study (%)****.*

| ***Food*** | ***Control group*** | | ***CNP group*** | | | |
| --- | --- | --- | --- | --- | --- | --- |
|  | ***Baseline*** | ***Change*** | ***Baseline*** | ***Change*** | ***Intervention effect (OR, 95% CI)*** | ***P-value*** |
| *Cereals (>3 times /3 days)* | *79.9* | *-10.8* | *78.5* | *-5.4* | *1.4 (1.1, 1.7)* | *0.009* |
| *Meat (>3 times /3 days)* | *59.0* | *-0.8* | *62.5* | *3.0* | *1.2 (1.0, 1.5)* | *0.091* |
| *Vegetables (>3 times /3 days)* | *65.7* | *-0.4* | *68.4* | *-1.6* | *0.9 (0.7, 1.1)* | *0.521* |
| *Fruits (≥1 time /3 days)* | *46.0* | *-1.9* | *45.0* | *5.0* | *1.4 (1.1, 1.7)* | *0.003* |
| *Dairy (≥1 time /3 days)* | *68.8* | *-4.5* | *71.4* | *-4.0* | *1.0 (0.8, 1.2)* | *0.832* |
| *Eggs (≥1 time /3 days)* | *74.3* | *4.2* | *78.4* | *-0.1* | *0.8 (0.7, 1.0)* | *0.031* |
| *Fish and shellfish (≥1 time /3 days)* | *54.2* | *-2.5* | *57.1* | *-8.4* | *0.7 (0.6, 0.9)* | *0.005* |
| *Fungi and algae (≥1 time /3 days)* | *35.3* | *-0.5* | *35.2* | *-0.6* | *1.0 (1.2, 1.2)* | *0.702* |
| *Dried legumes (≥1 time /3 days)* | *53.9* | *2.5* | *59.0* | *-8.3* | *0.6 (0.5, 0.7)* | *0.001* |
| *Nuts and seeds (≥1 time /3 days)* | *16.3* | *-3.4* | *12.3* | *-2.7* | *0.9 (0.7, 1.2)* | *0.665* |
| *Snack (≥1 time /3 days)* | *18.4* | *-2.1* | *20.1* | *-4.2* | *0.8 (0.7, 1.1)* | *0.162* |
| *Fast foods (≥1 time /3 days)* | *70.5* | *-5.8* | *70.1* | *-5.6* | *1.0 (0.9, 1.2)* | *0.841* |
| *Beverages (≥1 time /3 days)* | *34.7* | *-10.5* | *31.2* | *-9.1* | *1.1(0.9, 1.4)* | *0.561* |
| *Sugars and preserves (≥1 time /3 days)* | *23.5* | *-3.5* | *20.7* | *-3.0* | *1.0 (0.8,1.2)* | *0.839* |

*CI, Confidence Interval; CNP, comprehensive intervention including nutrition education and physical activity; OR, Odds Ratio.*

*A generalized linear mixed model was used for comparison, the effect was adjusted for sex, age, parental education level and family income level.*

***Table 7. The proportion of children consuming different food groups in NE or PA study (%)****.*

| ***Food*** | ***Control group*** | | ***NE group*** | | | | ***PA group*** | | | |
| --- | --- | --- | --- | --- | --- | --- | --- | --- | --- | --- |
|  | ***Baseline*** | ***Change*** | ***Baseline*** | ***Change*** | ***Intervention effect (OR, 95% CI)*** | ***P*** | ***Baseline*** | ***Change*** | ***Intervention effect (OR, 95% CI)*** | ***P-value*** |
| *Cereals (>3 times /3 days)* | *72.1* | *5.8* | *85.5* | *5.7* | *0.8 (0.3, 1.2)* | *0.634* | *85.1* | *2.0* | *0.8 (0.3, 1.2)* | *0.650* |
| *Meat (>3 times /3 days)* | *34.4* | *-4.1* | *38.6* | *8.8* | *1.5 (0.8,3)* | *0.199* | *28.4* | *-1.3* | *1.2 (0.6, 2.5)* | *0.557* |
| *Vegetables (>3 times /3 days)* | *51.6* | *-6.5* | *67.6* | *13.7* | *2.2 (1.2, 4.5)* | *0.014* | *73.9* | *-16.5* | *0.6 (0.3, 1.0)* | *0.061* |
| *Fruits (≥1 time /3 days)* | *36.1* | *6.5* | *59.1* | *-20.2* | *0.3 (0.2, 0.6)* | *0.001* | *49.2* | *-10.3* | *0.5 (0.3, 0.9)* | *0.023* |
| *Dairy (≥1 time /3 days)* | *78.7* | *-13.9* | *74.4* | *-5.6* | *1.4 (0.7, 2.5)* | *0.459* | *69.3* | *-11.5* | *1.0 (0.6,2.2)* | *0.837* |
| *Eggs (≥1 time /3 days)* | *76.2* | *0.8* | *93.2* | *-2.6* | *0.5 (4.5, 1.2)* | *0.123* | *80.9* | *2.9* | *0.9 (2.2, 2.0)* | *0.865* |
| *Fish and shellfish (≥1 time /3 days)* | *18.0* | *2.5* | *25.3* | *-1.7* | *0.8 (0.4, 1.7)* | *0.527* | *21.1* | *-3.3* | *0.7 (0.3, 1.4)* | *0.260* |
| *Fungi and algae (≥1 time /3 days)* | *18.0* | *3.3* | *34.1* | *25.6* | *2.5 (1.1, 5.0)* | *0.024* | *26.7* | *-0.6* | *0.7 (0.3, 1.4)* | *0.275* |
| *Dried legumes (≥1 time /3 days)* | *38.5* | *18.9* | *60.5* | *2* | *0.5 (0.2, 0.8)* | *0.011* | *51.8* | *5.3* | *0.6 (0.3, 1.0)* | *0.048* |
| *Nuts and seeds (≥1 time /3 days)* | *10.7* | *-3.3* | *17.0* | *5.4* | *2.2 (1.2, 6.1)* | *0.103* | *16.8* | *-4.3* | *0.9 (0.3, 2.5)* | *0.875* |
| *Snack (≥1 time /3 days)* | *15.6* | *-8.2* | *42.9* | *-30.7* | *0.3 (0.1, 0.7)* | *0.006* | *21.5* | *-13.6* | *0.7 (0.3, 1.8)* | *0.44* |
| *Fast foods (≥1 time /3 days)* | *58.2* | *9.0* | *70.5* | *-13.1* | *0.4 (0.2, 0.7)* | *0.002* | *56.8* | *-7.3* | *0.5 (0.3, 0.9)* | *0.027* |
| *Beverages (≥1 time /3 days)* | *25.4* | *-1.6* | *41.8* | *-7.1* | *0.9 (0.5, 1.8)* | *0.697* | *36.6* | *-7.2* | *0.8 (0.4, 1.7)* | *0.641* |
| *Sugars and preserves (≥1 time /3 days)* | *13.1* | *4.9* | *15.1* | *0.2* | *0.7 (0.3, 1.5)* | *0.309* | *13.2* | *-0.7* | *0.6 (0.2, 1.4)* | *0.189* |

*CI, Confidence Interval; NE, nutrition education intervention; OR, Odds Ratio; PA, physical activity intervention.*

*A generalized linear mixed model was used for comparison, the effect was adjusted for sex, age, parental education level and family income level.*” ***L495-530, L647-670.***

Reviewer #3: (No Response)
