# Supplementary material for: The effect of comprehensive intervention for childhood obesity on dietary diversity among younger children: Evidence from a school-based randomized controlled trial in China
Source: PLoS One. 2020 Jul 17;15(7):e0235951. doi: 10.1371/journal.pone.0235951 (PMC7367455; doi:10.1371/journal.pone.0235951)
Supplement: Attachment — Submitted filename: Response to Reviewers.docx [file pone.0235951.s007.docx]

6 Jun 2020

Dear Dr. Seth Adu-Afarwuah, Editors and Reviewers:

We very much appreciate your thoughtful comments on our manuscript (PONE-D-19-24892R2, “The effect of comprehensive intervention for childhood obesity on dietary diversity among younger children: Evidence from a school-based randomized controlled trial in China”). We have carefully considered each comment and responded point-by-point below. We have made corresponding changes in the manuscript with tracked changes highlighting both additions and deletions.

In addition, we have carefully checked the format requirement and revised the manuscript according to the requirement of *PLOS ONE*.

We believe that your comments and our responses to them have improved our manuscript considerably, and we hope you find our revised manuscript suitable for publication.

Sincerely,

Guansheng Ma Prof.

Department of Nutrition and Food Hygiene,

School of Public Health, Peking University,

38 Xue Yuan Road,

Beijing, China.

Email: [mags@bjmu.edu.cn](mailto:mags@bjmu.edu.cn)

<Tel:13801080336>,

Fax: +86 10 82105184.

Guansheng Ma Prof.

Department of Nutrition and Food Hygiene,

School of Public Health, Peking University,

38 Xue Yuan Road,

Beijing, China.

Email: [mags@bjmu.edu.cn](mailto:mags@bjmu.edu.cn)

<Tel:13801080336>,

Fax: +86 10 82105184.

Responses to Reviewer #2:

We much appreciate the valuable suggestions from the reviewer, which make the manuscript have much improvement.

**2.1** In statistical analysis section, the authors stated that Pij in equation (2) is the change of probability of an event from baseline to end for child i at level j. I disagree with this statement as the logit link is suitable to a binary outcome (Yes or No), which cannot be calculated as change from baseline for each participant. Using same notations as equation (1), the authors should say that the same linear equation was applied to binary outcomes with a logit link as part of generalized linear models. With no time effect in the model, the sentence "The effect of the intervention was evaluated by testing the interaction term between time and treatment" should be removed.
**Response:** We appreciate the valuable suggestions from the review expert. We agree with the expert and delete the sentence “*The effect of the intervention was evaluated by testing the interaction term between time and treatment*” to make the describing more rigorous.

“*The treatment effects were estimated using equation (1) for DDS and FVS and equation (2) for proportions of children consuming certain foods.*

*Y_ij_ =* *γ_00_ +β_0_Cluster+ β_01_Treat + β_1_X_1ij_ + β_Ƥ_X_Ƥij_ +ε_ij_	(1)*

*Logit (P_ij_) = γ_00_ +β_0_Cluster + β_01_Treat + β_1_X_1ij_ + β_Ƥ_X_Ƥij_ +ε_ij_	(2)*

*In equation (1), Y_ij_ is the outcome of change from baseline to end for child i at level j; γ_00_ is the intercept parameter; Cluster is random effect indicator; β_01_ reveals the intervention effect; Treat is an indicator that equals 1 if the student is in the treatment arm and 0 otherwise; X_1ij_ is the outcome at baseline and X_Ƥij_ is a vector of control variables at baseline. The same linear equation (2) was applied to binary outcomes with a logit link as part of generalized linear models.*” ***Page 13, L429-438, in revised manuscript with track changes.***

**2.2** In the results, the authors have used the difference in change of proportions between two groups to quantify the intervention effect on food group consumption, rather than the estimated odds ratio and 95% CI. Although I understand that the difference in proportions is more intuitive to interpret, the adjusted model estimate is more robust which has taken into account baseline covariates and random cluster effect.
**Response:** We appreciated the valuable suggestion from the reviewer and added the estimated odds ratio and 95% CI in the results description.

*“The effects of the three interventions on food group consumption changes are shown in Table 6 and 7. Compared with the control group, the proportions of children consuming cereals (> 3 times/3 days) and fruits (≥ 1 time/3 days) increased significantly, and Eggs (≥1 time /3 days), fish (≥1 time /3 days) and dried legumes (≥1 time /3 days) decreased significantly in the CNP group, with changes of 5.4 (-5.4 vs. -10.8 for treat vs. control; Odds Ratio (OR) : 1.4 (95% CI: 1.1,1.7); p = 0.009) and 6.9 (5.0 vs. -1.9; OR: 1.4 (95% CI: 1.1,1.7); p = 0.003) percent, -4.3 (-0.1 vs. 4.2 for treat vs. control; OR: 0.8 (95% CI: 0.7,1.0); p = 0.031), -5.9 (-8.4 vs. -2.5; OR: 0.7 (95% CI: 0.6,0.9); p = 0.005) and -10.8 (-8.3 vs. 2.5; OR: 0.6 (95% CI: 0.5,0.7); p < 0.001) percent, respectively. The proportion changes among the NE group in the consumption of vegetables(> 3 time /3 days), fruits (≥ 1 time/3 days), Fungi and algae (≥1 time /3 days), dried legumes (≥ 1 time/3 days), snack (≥ 1 time/3 days) and fast foods (≥1 time / 3 days) were 20.2 (13.7 vs. -6.5 for treat vs. control; OR: 2.2 (95% CI: 1.2,4.5); p = 0.014), -26.7 (-20.2 vs. 6.5; OR: 0.3 (95% CI: 0.2,0.6); p = 0.001), 22.3 (25.6 vs. 3.3; OR: 2.5 (95% CI: 1.1,5.0); p = 0.024), -16.9 (2.0 vs. 18.9; OR: 0.5 (95% CI: 0.2,0.8); p = 0.011), -22.5 (-30.7 vs. -8.2; OR: 0.3 (95% CI: 0.1,0.7); p = 0.006) and -22.1 (-13.1 vs. 9.0; OR: 0.4 (95% CI: 0.2,0.7); p = 0.002) percent, respectively. The PA group showed no significant increases but significant decreases (or relative decreases) in the proportions of children consuming fruits (≥ 1 time/3 days), dried legumes (≥ 1 time/3 days) and fast foods (≥ 1 time/3 days), which were -16.8 (-10.3 vs. 6.5 for treat vs. control; OR: 0.5 (95% CI: 0.3,0.9); p = 0.023), -13.6 (5.3 vs. 18.9; OR: 0.6 (95% CI: 0.3,1.0); p = 0.048) and -16.3 (-7.3 vs. 9.0; OR: 0.5 (95% CI: 0.3,0.9); p = 0.027) percent compared with the control group, respectively.”* ***Page 22-23, L850-984, in revised manuscript with track changes.***

**2.3** Please follow the CONSORT 2010 Explanation and Elaboration in reporting tables and figures, with the extension to cluster randomised trials.

**Response:** We appreciate the suggestion and revised the tables by supplying the information of the number of participants with the event in Table 6 and Table 7 following the CONSORT 2010 Explanation and Elaboration.

*“****Table 6. The proportion of children consuming different food groups in CNP study****.*

| ***Food*** | ***Control group*** | | ***CNP group*** | | | |
| --- | --- | --- | --- | --- | --- | --- |
|  | ***Baseline (n (%))*** | ***Change (n (%))*** | ***Baseline (n (%))*** | ***Change (n (%))*** | ***Intervention effect (OR, 95% CI)*** | ***P-value*** |
| *Cereals (>3 times /3 days)* | *1530 (79.9)* | *-206 (-10.8)* | *1690 (78.5)* | *-117 (-5.4)* | *1.4 (1.1, 1.7)* | *0.009* |
| *Meat (>3 times /3 days)* | *1130 (59.0)* | *-14 (-0.8)* | *1345 (62.5)* | *65 (3.0)* | *1.2 (1.0, 1.5)* | *0.091* |
| *Vegetables (>3 times /3 days)* | *1259 (65.7)* | *-8 (-0.4)* | *1473 (68.4)* | *-35 (-1.6)* | *0.9 (0.7, 1.1)* | *0.521* |
| *Fruits (≥1 time /3 days)* | *881 (46.0)* | *-37 (-1.9)* | *969 (45.0)* | *108 (5.0)* | *1.4 (1.1, 1.7)* | *0.003* |
| *Dairy (≥1 time /3 days)* | *1318 (68.8)* | *-86 (-4.5)* | *1536 (71.4)* | *-85 (-4.0)* | *1.0 (0.8, 1.2)* | *0.832* |
| *Eggs (≥1 time /3 days)* | *1424 (74.3)* | *81 (4.2)* | *1688 (78.4)* | *-3 (-0.1)* | *0.8 (0.7, 1.0)* | *0.031* |
| *Fish and shellfish (≥1 time /3 days)* | *1039 (54.2)* | *-48 (-2.5)* | *1231 (57.1)* | *-181 (-8.4)* | *0.7 (0.6, 0.9)* | *0.005* |
| *Fungi and algae (≥1 time /3 days)* | *676 (35.3)* | *-10 (-0.5)* | *758 (35.2)* | *-14 (-0.6)* | *1.0 (1.2, 1.2)* | *0.702* |
| *Dried legumes (≥1 time /3 days)* | *1032 (53.9)* | *48 (2.5)* | *1270 (59.0)* | *-179 (-8.3)* | *0.6 (0.5, 0.7)* | *0.001* |
| *Nuts and seeds (≥1 time /3 days)* | *312 (16.3)* | *-64 (-3.4)* | *265 (12.3)* | *-59 (-2.7)* | *0.9 (0.7, 1.2)* | *0.665* |
| *Snack (≥1 time /3 days)* | *352 (18.4)* | *-39 (-2.1)* | *432 (20.1)* | *-90 (-4.2)* | *0.8 (0.7, 1.1)* | *0.162* |
| *Fast foods (≥1 time /3 days)* | *1349 (70.5)* | *-109 (-5.8)* | *1510 (70.1)* | *-120 (-5.6)* | *1.0 (0.9, 1.2)* | *0.841* |
| *Beverages (≥1 time /3 days)* | *665 (34.7)* | *-202 (-10.5)* | *672 (31.2)* | *-195 (-9.1)* | *1.1(0.9, 1.4)* | *0.561* |
| *Sugars and preserves (≥1 time /3 days)* | *450 (23.5)* | *-66 (-3.5)* | *445 (20.7)* | *-64 (-3.0)* | *1.0 (0.8,1.2)* | *0.839* |

*CI, Confidence Interval; CNP, comprehensive intervention including nutrition education and physical activity; OR, Odds Ratio.*

*A generalized linear mixed model was used for comparison, the effect was adjusted for sex, age, parental education level and family income level.*

***Table 7. The proportion of children consuming different food groups in NE or PA study (%)****.*

| ***Food*** | ***Control group*** | | ***NE group*** | | | | ***PA group*** | | | |
| --- | --- | --- | --- | --- | --- | --- | --- | --- | --- | --- |
|  | ***Baseline (n (%))*** | ***Change (n (%))*** | ***Baseline (n (%))*** | ***Change (n (%))*** | ***Intervention effect (OR, 95% CI)*** | ***P*** | ***Baseline (n (%))*** | ***Change (n (%))*** | ***Intervention effect (OR, 95% CI)*** | ***P-value*** |
| *Cereals (>3 times /3 days)* | *88 (72.1)* | *7 (5.8)* | *301 (85.5)* | *20 (5.7)* | *0.8 (0.3, 1.2)* | *0.634* | *258 (85.1)* | *6 (2.0)* | *0.8 (0.3, 1.2)* | *0.650* |
| *Meat (>3 times /3 days)* | *42 (34.4)* | *-5 (-4.1)* | *136 (38.6)* | *31 (8.8)* | *1.5 (0.8,3)* | *0.199* | *86 (28.4)* | *4 (1.3)* | *1.2 (0.6, 2.5)* | *0.557* |
| *Vegetables (>3 times /3 days)* | *63 (51.6)* | *-8 (-6.5)* | *238 (67.6)* | *48 (13.7)* | *2.2 (1.2, 4.5)* | *0.014* | *224 (73.9)* | *-50 (-16.5)* | *0.6 (0.3, 1.0)* | *0.061* |
| *Fruits (≥1 time /3 days)* | *44 (36.1)* | *8 (6.5)* | *208 (59.1)* | *-71 (-20.2)* | *0.3 (0.2, 0.6)* | *0.001* | *149 (49.2)* | *-31 (-10.3)* | *0.5 (0.3, 0.9)* | *0.023* |
| *Dairy (≥1 time /3 days)* | *96 (78.7)* | *-17 (-13.9)* | *262 (74.4)* | *-20 (-5.6)* | *1.4 (0.7, 2.5)* | *0.459* | *210 (69.3)* | *-35 (-11.5)* | *1.0 (0.6,2.2)* | *0.837* |
| *Eggs (≥1 time /3 days)* | *93 (76.2)* | *1 (0.8)* | *328 (93.2)* | *-9 (-2.6)* | *0.5 (4.5, 1.2)* | *0.123* | *245 (80.9)* | *9 (2.9)* | *0.9 (2.2, 2.0)* | *0.865* |
| *Fish and shellfish (≥1 time /3 days)* | *22 (18.0)* | *3 (2.5)* | *88 (25.3)* | *-5 (-1.7)* | *0.8 (0.4, 1.7)* | *0.527* | *64 (21.1)* | *-10 (-3.3)* | *0.7 (0.3, 1.4)* | *0.260* |
| *Fungi and algae (≥1 time /3 days)* | *22 (18.0)* | *4 (3.3)* | *120 (34.1)* | *90 (25.6)* | *2.5 (1.1, 5.0)* | *0.024* | *81 (26.7)* | *-2 (-0.6)* | *0.7 (0.3, 1.4)* | *0.275* |
| *Dried legumes (≥1 time /3 days)* | *47 (38.5)* | *23 (18.9)* | *213 (60.5)* | *7 (2.0)* | *0.5 (0.2, 0.8)* | *0.011* | *157 (51.8)* | *16 (5.3)* | *0.6 (0.3, 1.0)* | *0.048* |
| *Nuts and seeds (≥1 time /3 days)* | *13 (10.7)* | *-4 (-3.3)* | *60 (17.0)* | *19 (5.4)* | *2.2 (1.2, 6.1)* | *0.103* | *51 (16.8)* | *-13 (-4.3)* | *0.9 (0.3, 2.5)* | *0.875* |
| *Snack (≥1 time /3 days)* | *19 (15.6)* | *-10 (-8.2)* | *151 (42.9)* | *-108 (-30.7)* | *0.3 (0.1, 0.7)* | *0.006* | *65 (21.5)* | *-41 (-13.6)* | *0.7 (0.3, 1.8)* | *0.44* |
| *Fast foods (≥1 time /3 days)* | *71 (58.2)* | *11 (9.0)* | *248 (70.5)* | *-46 (-13.1)* | *0.4 (0.2, 0.7)* | *0.002* | *172 (56.8)* | *-22 (-7.3)* | *0.5 (0.3, 0.9)* | *0.027* |
| *Beverages (≥1 time /3 days)* | *31 (25.4)* | *-2 (-1.6)* | *147 (41.8)* | *-25 (-7.1)* | *0.9 (0.5, 1.8)* | *0.697* | *111 (36.6)* | *-22 (-7.2)* | *0.8 (0.4, 1.7)* | *0.641* |
| *Sugars and preserves (≥1 time /3 days)* | *16 (13.1)* | *6 (4.9)* | *53 (15.1)* | *1 (0.2)* | *0.7 (0.3, 1.5)* | *0.309* | *40 (13.2)* | *-2 (-0.7)* | *0.6 (0.2, 1.4)* | *0.189* |

*CI, Confidence Interval; NE, nutrition education intervention; OR, Odds Ratio; PA, physical activity intervention.*

*A generalized linear mixed model was used for comparison, the effect was adjusted for sex, age, parental education level and family income level*.” ***Page23-26, L986-933, in revised manuscript with track changes.***
